# Antibacterial spirooxindole alkaloids from *Penicillium brefeldianum* inhibit dimorphism of pathogenic smut fungi

**DOI:** 10.3389/fmicb.2022.1046099

**Published:** 2022-11-14

**Authors:** Huajun Shi, Jinyan Jiang, Hang Zhang, Haimei Jiang, Zijie Su, Dandan Liu, Ligang Jie, Fei He

**Affiliations:** ^1^School of Traditional Chinese Medicine, Southern Medical University, Guangzhou, China; ^2^Department of Applied Biological Chemistry, Graduate School of Agricultural and Life Sciences, The University of Tokyo, Tokyo, Japan; ^3^Zhujiang Hospital, Southern Medical University, Guangzhou, China

**Keywords:** *Penicillium brefeldianum*, spirooxindole diketone piperazine, absolute configuration, antibacterial activities, fungal secondary metabolites

## Abstract

Three new antibacterial spirooxindole alkaloids, spirobrefeldins A–C (1–3), together with four known analogs, spirotryprostatin M (4), spirotryprostatin G (5), 12β-hydroxyverruculogen TR-2 (6), and 12α-hydroxyverruculogen TR-2 (7), were isolated from terrestrial fungus *Penicillium brefeldianum.* All the new compounds were elucidated extensively by the interpretation of their NMR (1D and 2D) spectra and high-resolution mass data, and their absolute configurations were determined by computational chemistry and CD spectra. The absolute configurations of spiro carbon C-2 in spirotryprostatin G (5) and spirotryprostatin C in literature were reported as *S*, which were revised to *R* based on experimental and calculated CD spectra. All the compounds were evaluated for their antimicrobial activities toward *Pseudomonas aeruginosa* PAO1, *Dickeya zeae* EC1, *Staphylococcus epidermidis*, *Escherichia coli*, and *Sporisorium scitamineum*. Compound 7 displayed moderate inhibitory activity toward dimorphic switch of pathogenic smut fungi *Sporisorium scitamineum* at 25 μM. Compounds 3 and 6 showed weak antibacterial activities against phytopathogenic bacterial *Dickeya zeae* EC1 at 100 μM.

## Introduction

Microbes have been considered to be a significant source of bioactive secondary metabolites for drugs (Demain and Sanchez, 2009; Newman, 2021). Fungi as one of the widest phyla of organisms spread all over the world inhabiting all substrates and climate conditions. It is estimated that at least 18,000 species of fungi have been described (Marin-Felix et al., 2017). Fungi are also well known to produce secondary metabolites, such as terpenoids, alkaloids, macrolides, polyketides, and pigments, with diverse significant biological activities such as anti-tumor, antioxidant, anti-inflammatory, antimicrobial, and anticancer, which could be widely used in the pharmaceutical and agricultural industries (Bills and Gloer, 2016; Keller, 2019; Steele et al., 2019; Adeleke and Babalola, 2021; Tiwari and Bae, 2022; Wen et al., 2022). Penicillin is probably the best known β-lactam antibiotic drug made by fungi strains. Besides, Lovastatin, which is used to lower LDL cholesterol, and Cyclosporine, which suppresses the immune system activity and treats some autoimmune diseases, are both well-known fungal secondary metabolite-derived drugs (Schueffler and Anke, 2014).

Spirooxindole ring is widely distributed in various bioactive natural products and has been used as a promising pharmacophore in drug discovery (Rottmann et al., 2010; Ye et al., 2016). These structures feature a spiro ring at the C-2 or C-3 position of the oxindole core with a heterocyclic skeleton. Interestingly, spirooxindole alkaloids with both *R* and *S* absolute configurations at the C-3 position were reported in the literature, such as paraherquamide N (3*R*) (Blanchflower et al., 1993), notoamide B (3*R*) (Kato et al., 2007), cyclopiamine A (3*R*) (Bond et al., 1979), and brevianamide X (3*S*) (Paterson et al., 1987), chrysogenamide A (3*S*) (Lin et al., 2008), citrinalin A (3*S*) (Tsuda et al., 2004), and citrinadin C (3*S*) (Jiang et al., 2022), while the absolute configurations of spiro carbon at C-2 position showed only *S* absolute configuration, such as spirotryprostatin M (Lin et al., 2020), spirotryprostatin G (Zhang et al., 2019), and spirotryprostatin C (Wang et al., 2008). Many spirooxindole alkaloids have been found to show significant biological activity, including anticancer, insecticidal, cytotoxic, and antibacterial activities (Tsukamoto et al., 2010; Kagiyama et al., 2016; Klas et al., 2018). The unique structural features and diverse biological activities of spirooxindole alkaloids have brought great interest and challenge to chemists for total synthesis and biosynthesis (Greshock et al., 2007; Bian et al., 2013; Mercado-Marin et al., 2014; Liu et al., 2021).

**FIGURE 1:**
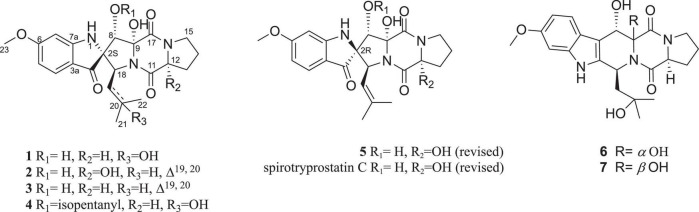
Compounds 1–7 isolated and identified from *Penicillium brefeldianum* and revised structures of compounds 5 and spirotryprostatin C.

In our continuing investigation for new pharmacologically active secondary metabolites from microbes (He et al., 2012, 2013a,2013b; Zhang et al., 2016; Wu et al., 2017; Jiang et al., 2022), the bioactive natural products of *Penicillium brefeldianum* have been studied. Three new spirooxindole alkaloids, sprirobrefeldins A–C (1–3), together with four known ones, spirotryprostatin M (4) (Supplementary Figures S28, S29), spirotryprostatin G (**5**), 12β-hydroxyverruculogen TR-2 (6) (Supplementary Figures S32, S33) (Li et al., 2012), and 12α-hydroxyverruculogen TR-2 (7) (Supplementary Figures S34, S35) (Li et al., 2012), were isolated (Figure 1). The absolute configurations of spiro carbon at C-2 position in spirotryprostatin G (5) and spirotryprostatin C in literature were reported as *S*, which were revised to *R* based on experimental and calculated CD spectra. This is the first report of spirooxindoles with spiro carbon at the C-2 position that have both *S* and *R* configurations. All the compounds were evaluated for their antimicrobial activities toward *Pseudomonas aeruginosa* PAO1, *Dickeya zeae* EC1, *Staphylococcus epidermidis*, *Escherichia coli*, and *Sporisorium scitamineum*. Compound 7 displayed moderate inhibitory activity toward dimorphic switch of *Sporisorium scitamineum*, with an MIC value of 25 μM. Around 100 M, compounds 3 and 6 showed weak antibacterial activities against phytopathogenic bacterial *Dickeya zeae*.

## Materials and methods

### General experimental procedures

FT-IR spectrometer (Affinity-1, Shimadzu) was used to measure IR spectra. Optical rotations were measured in a polarimeter (MCP 300, Anton Paar) at 25°C. U-2910 spectrometer (Hitachi) was used to record UV spectra. Advance 600 spectrometer (Bruker) was used to measure ^1^H NMR (600 MHz) and ^13^C NMR (150 MHz). Esquire 3000 plus spectrometer (Bruker) was used to measure ESIMS spectra. A micro TOF-QII mass spectrometer (Bruker) was used to record HRESIMS data. Sephadex LH-20 gel (Amersham Pharmacia) and silica gel (100–200 mesh and 200–300 mesh; Qingdao Marine Chemicals) were used in column chromatography. Analytical and preparative HPLC was performed on a Shimadzu Prominence system. Circular Dichroism Spectrometer (V100) was used to measure CD spectra.

### Fungal materials

The strain *P. brefeldianum* used in this project was isolated from soil samples collected in the Tengchong forest of Yunnan province, China. The isolate was identified by Miss Jinyan Jiang based on the morphology and sequence analysis of the ITS region of the rDNA (GenBank Accession Number is 138263), and a voucher specimen (*Penicillium brefeldianum* SMU008) was stored in the School of Chinese Medicine, Southern Medical University.

### Fermentation and extraction

The fresh mycelia of *Penicillium brefeldianum* were initially grown on the PDA medium at 28°C (72 h). Small pieces of Agar plugs were selected to inoculate 10 Erlenmeyer flasks (500 mL) each containing 200 mL of PDB, and were cultured for 5 days (shake, 150 rpm, 28°C). The seed culture was then inoculated into 50 × 500 mL conical flasks on rice solid medium (80 g rice, 120 mL of filtered water) for 28 days at room temperature. The fermented solid cultures were then extracted fully with ethyl acetate to yield 12-gram crude extract.

### Isolation and purification

The crude extract had been chromatographed on silica using elution system with CHCl_3_/MeOH (v/v, 100:0, 95:5, 9:1, 8:2, 1:1, and 0:100) to give six crude parts (Fraction A–Fraction F). Fr.D was further purified to afford five subfractions (Fr.D-1 to Fr.D-5) using silica column chromatography eluting with CH_2_Cl_2_/MeOH. Fr.D-1 was isolated by Sephadex LH-20 using CH_2_Cl_2_/MeOH (v/v, 1:1) to obtain five subfractions. Then Fr. D-1-2 was separated on ODS column with MeOH/H_2_O (10%, 30%, 50%, 70%, 80%, 100%) to obtain six fractions (Fr.D-1-2-1 to Fr.D-1-2-6). Eight fractions (Fr.D-1-2-4-1 to Fr.D-1-2-4-8) were obtained from Fr.D-1-2-4 by p-TLC (CHCl_3_-acetone, 2:1 v/v). 1 (5 mg), 2 (5 mg), and 3 (6 mg) were separated from Fr.D-1-2-4-7 by p-HPLC (v/v, 45% MeOH/H_2_O, 3.0 mL/min with retention time of 20 min, 25 min, 29 min, respectively. Fr.D-1-2-4-4 was further isolated by p-HPLC (v/v, 50% MeOH/H_2_O, 3.0 mL/min) to obtain 5 (4 mg) with retention time of 18 min. Fr. D-1-2-4-5 was further purified by HPLC (v/v, 30% ACN/H_2_O, 3.0 mL/min) to obtain 6 (12 mg) with a retention time of 19 min. Fr.D-1-2-4-6 was purified by HPLC (v/v, 40% MeOH/H_2_O, 3.0 mL/min) to obtain 7 (5 mg) with a retention time of 29 min. Fr.C was further purified by silica C.C. with hexane/EtOAc system to afford five subfractions (Fr.C-1 to Fr.C-5). Then Fr.C-5 was separated by CH_2_Cl_2_/MeOH to afford seven subfractions (Fr.C-5-1 to Fr.C-5-7). Seven fractions (Fr.C-5-3-1 to Fr.C-5-3-7) were obtained from Fr.C-5-3 by p-TLC (v/v, CHCl_3_/acetone, 4:1). 4 (10 mg) was obtained from Fr.D-5-3-5 by p-HPLC (v/v, 60% MeOH/H_2_O, 3.0 mL/min) with a retention time of 30 min.

Spirobrefeldin A (1): pale yellow powder; UV (MeOH) λ_*max*_ (log ε) 203 (4.08), 224 (4.12), 249 (4.08), 281 (3.87), 374 (3.39) nm. CD (MeOH) λ_*max*_ (Δε) 200 (+ 21.2), 227 (− 27.4), 283 (+ 6.0), 320 (− 4.7), 353 (+ 0.8), 390 (− 2.9) nm; HRESIMS *m*/*z* 444.1772 [M − H]^–^, (calculated for C_22_H_25_N_3_O_7_, 444.1776); IR (neat) ν_*max*_ 3,432, 2,941, 1,668, 1,662, 1,614, 1,456, 1,303, 1,215, and 1,024 cm^–1^; [α]25 D − 81.2 (*c* 0.09, MeOH) (Supplementary Figures S1–S9).

Spirobrefeldin B (2): amorphous yellow powder; UV (MeOH) λ_*max*_ (log ε) 203 (4.04), 225 (3.97), 248 (3.83), 284 (3.69), 374 (3.16) nm; CD (MeOH) λ_*max*_ (Δε) 200 (+ 15.8), 231 (− 64.6), 256 (+ 16.3), 282 (+ 6.5), 313 (− 20.2), 366 (+ 4.3) nm; HRESIMS *m*/*z* 442.1615 [M − H]^–^, (calculated for C_22_H_24_N_3_O_7_, 442.1620); IR (neat) ν_*max*_ 3,344, 3,334, 1,681, 1,662, 1,614, 1,456, 1,396, 1,213, and 1,024 cm^–1^; [α]25 D − 69.1 (*c* 0.08, MeOH) (Supplementary Figures S10–S18).

Spirobrefeldin C (3): amorphous yellow powder; UV (MeOH) λ_*max*_ (log ε) 204 (4.23), 227 (4.23), 248 (4.15), 284 (4.01), 375 (3.50) nm; CD (MeOH) λ_*max*_ (Δε) 229 (− 32.3), 255 (+ 8.9), 282 (+ 2.5), 315 (− 10.1), 361 (+ 2.2) nm; HRESIMS *m*/*z* 426.1671 [M − H]^–^, (calculated for C_22_H_24_N_3_O_6_, 426.1671); IR (neat) ν_*max*_ 3,344, 3,334, 1,670, 1,610, 1,456, 1,309, 1,213, and 1,022 cm^–1^; [α]25 D − 151.1 (*c* 0.08, MeOH) (Supplementary Figures S19–S27).

Spirotryprostatin G (5): amorphous yellow powder; CD (MeOH) λ_*max*_ (Δε) 201 (+ 34.8), 223 (− 9.1), 252 (− 15.8), 283 (− 3.2), 307 (+ 6.4), 387 (+ 3.7) nm; ESIMS *m*/*z* 442.10 [M − H]^–^; [α]25 D + 60.9 (*c* 0.1, MeOH) (Supplementary Figures S30, S31, S36–S38 and Supplementary Tables S1–S8).

### Antibacterial assay

The plant pathogenic smut fungi used in this assay is *Sporisorium scitamineum*, and tested compounds were dissolved in DMSO in different concentrations. MAT-1 and MAT-2 colonies were cultured in 5 mL of YEPSA overnight (28°C, 200 rpm), respectively. Then 1 mL of YEPSA medium (agar) with different concentrations of compounds was added to a 24-well plate. After that, 1 μL of the mixture of MAT-1 and MAT-2 was added to each well. The well without compounds was used as a negative control. The 24-well plate was incubated in a 28°C incubator for 2 days by observing hypha formation. MPA was used as a positive control in this assay (Zhong et al., 2018).

The bacterial strains used in this work (*Pseudomonas aeruginosa* PAO1, *Dickeya zeae* EC1, *Staphylococcus epidermidis*, and *Escherichia coli*.) were grown in LB medium at 30°C. Luria–Bertani (LB) medium (1 L contains 10 g trypeptone, 5 g yeast extract, and 10 g NaCl) was used to isolate biocontrol agents. Vancomycin and imipenem were used as positive control. Overnight cultured bacterial strains were diluted in fresh LB media to an OD_600_ of 0.1 in the absence or presence of compounds at different concentrations. The bacterial cells were grown in each well of a 96-well polystyrene plate at 37°C for 12 h with shaking. Then, a microplate reader was used to measure the absorbance of each well at 600 nm.

### Electronic circular dichroism calculations

The Gaussian 09 software was used to determine the absolute configurations of compounds 1, 2, and 5. Briefly, random conformational analyses were conducted on the basis of MMFF94 force fields before the relative configurations of compounds were determined by the NOESY spectra initially. The obtained conformers were optimized at the B3LYP/6-31G(d) level of time-dependent density functional theory (TDDFT) and followed by ECD calculations *via* TDDFT [B3LYP/6-31 + G(d), CPCM model = MeOH]. The ECD curves were generated by SpecDisv1.51 (Huo et al., 2018).

### Nuclear magnetic resonance calculation

The theoretical calculations were performed using Gaussian 16. The systematic random conformational analysis was performed in the Sybyl-X 2.0 program by using MMFF94s molecular force field and a global minima energy cutoff of 6 kcalmol-1. All the obtained conformers were further optimized using DFT at the B3LYP/6-31 G(d) level in the gas phase by using Gaussian 16 software. Harmonic vibrational frequencies were also performed to confirm no imaginary frequencies of the finally optimized conformers. On basis of the energies, conformers with a Boltzmann distribution > 1% were chosen. Gauge-independent atomic orbital (GIAO) calculations of 1H- and 13C-NMR chemical shifts were accomplished by DFT at the mPW1PW91/6-31 + G level in DMSO with the PCM solvent model in Gaussian 16 software. After Boltzmann weighing of the predicted chemical shift of each isomer, the linear correlation coefficients (R2), mean absolute deviation (MAD), root-mean-square deviation (RMSD), and corrected mean absolute deviation (CMAD) were calculated for the evaluation of the results. Moreover, the DP4 + parameters were calculated using the excel file provided by Grimblat et al. (2015) and Marcarino et al. (2022).

## Results and discussion

### Structure elucidation

**FIGURE 2:**
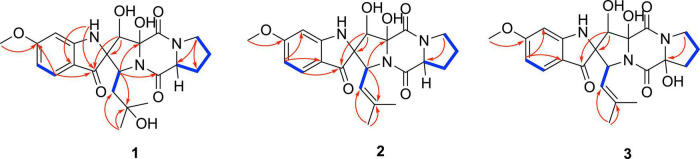
Key HMBC and COSY correlations of compounds 1–3.

Spirobrefeldin A (1) was isolated as an amorphous yellow powder and exhibited [M − H]^–^ ion peak at *m/z* 444.1772 (calcd. 444.1776) in the HRESIMS, associated with a molecular formula of C_22_H_26_N_3_O_7_, requiring 11 degrees of unsaturation. The IR spectrum of 1 showed absorption bands at 3344 (OH), 1670 (C = O), and 1610 (C = C) in the functional group region. The ^1^H NMR data of 1 (Table 1) showed signals of three aromatic protons at δ_*H*_ 7.26 (d, *J* = 8.6, H-4), 6.44 (d, *J* = 2.2, H-7), and 6.26 (dd, *J* = 8.5, 2.1, H-5), as well as one methoxyl group (δ_*H*_ 3.78), two methyl groups (δ_*H*_ 0.84, 0.99), and three methane protons at δ_*H*_ 4.14 (H-18), 4.38 (H-8), and 4.43 (H-12). The ^13^C and DEPT135 NMR spectra showed signals of 22 carbons, including three carbonyl carbons (δ_*C*_ 196.9, 169.5, 164.6), three sp^2^ quaternary carbons (δ_*C*_ 166.8, 163.5, 114.4), three sp^2^ methines (δ_*C*_ 124.9, 107.3, 94.4), three sp^3^ quaternary carbons (δ_*C*_ 85.2, 73.4, 68.3), three sp^3^ methines (δ_*C*_ 74.4, 59.8, 58.8), four sp^3^ methylene (δ_*C*_ 44.6, 38.5, 27.8, 22.7), and three methyl groups (δ_*C*_ 29.1, 30.2, 55.4). From the above observations and by comparison with NMR data from closely related structures, it was evident that 1 was similar to those of spirotryprostatin M (4), which suggested that 1 was spirooxindole diketone piperazine alkaloids. The above deduction was further confirmed by correlations from H-8 to C-2/C-3, N1-H to C-3/C-3a, H-18 to C-9/C-11/C-20, H-15 to C-12/C-13, and H-19 to C-21/C-22 in the HMBC spectrum, together with the ^1^H-^1^H COSY correlations, confirmed the connectivity of H-12/H-13/H-14/H-15 (Figure 2). Owing to the HRESIMS and ^13^C NMR data, it showed that the isopentenyl at C-18 in 4 disappeared and was substituted by a hydroxyl group in 1. Therefore, the planner structure of 1 was established.

**TABLE 1:** ^1^H and ^13^C NMR data (δ in ppm, *J* in Hz) for compounds 1, **2,** and 3^a^.

NO.	1		2		3	
	δ _*H*_^b^	δ _*C*_^c^	δ _H_	δ _C_	δ _H_	δ _C_
1-NH	7.09, s		7.19, s		7.16, s	
2		73.4 C		75.3 C		75.5 C
3		196.9 C		195.3 C		195.3 C
3a		114.4 C		113.3 C		113.4 C
4	7.26, d (8.6)	124.9 CH	7.27, d (8.6)	124.9 CH	7.26, d (8.6)	124.8 CH
5	6.26, dd (8.6, 2.2)	107.3 CH	6.28, dd (8.6, 2.2)	107.5 CH	6.28, dd (8.6, 2.2)	107.5 CH
6		166.8 C		167.1 C		166.9 C
7	6.44, d (2.2)	94.4 CH	6.46, d (2.2)	94.6 CH	6.46, d (2.2)	94.7 CH
7a		163.5 C		163.8 C		163.9 C
8	4.38, s	74.4 CH	4.43, s	74.4 CH	4.47, s	74.1 CH
9		85.2 C		85.1 C		85.4 C
11		169.5 C		167.0 C		168.6 C
12	4.43, dd (8.7, 6.8)	59.8 CH		88.8 C	4.40, dd (9.0, 7.2)	59.7 CH
13	1.90, m; 2.23, m	27.8 CH_2_	2.07, m	35.8 CH_2_	2.19, t (2.2); 1.83, m	27.8 CH_2_
14	1.85, m; 1.93, m	22.7 CH_2_	1.93, m	20.2 CH_2_	1.86, m	22.6 CH_2_
15	3.34, dt (11.6, 7.7)	44.6 CH_2_	3.48, m	44.5 CH_2_	3.46, m	44.5 CH_2_
17		164.6 C		165.6 C		164.8 C
18	4.14, dd (7.6, 1.8)	58.8 CH	4.63, d (9.5)	61.0 CH	4.61, d (9.6)	60.7 CH
19	1.37, dd (14.4, 1.8) 2.60, dd (14.4, 7.6)	38.5 CH_2_	4.99, m	120.9 CH	4.93, dt (9.6, 1.4)	121.1 CH
20		68.3 C		133.7 C		133.6 C
21	0.99, s	30.2 CH_3_	1.30, s	18.0 CH_3_	1.38, d (1.4)	17.9 CH_3_
22	0.84, s	29.1 CH_3_	1.58, s	25.4 CH_3_	1.57, d (1.4)	25.4 CH_3_
23	3.78, s	55.4 CH_3_	3.80, s	55.4 CH_3_	3.79, s	55.4 CH_3_
8-OH	5.63, s				5.64, s	
9-OH	6.95, s				7.05, s	
20-OH	4.06, s					

^a^Recorded in DMSO-*d*_6._^b^Recorded at 600 MHz. ^c^Recorded at 150 MHz.

**FIGURE 3:**
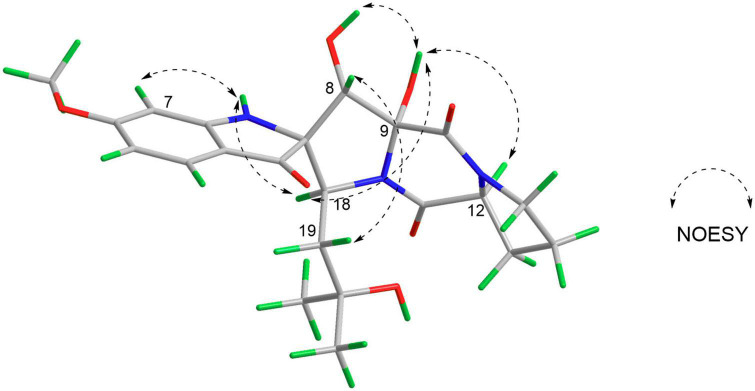
Key NOESY correlations of compound 1.

**FIGURE 4:**
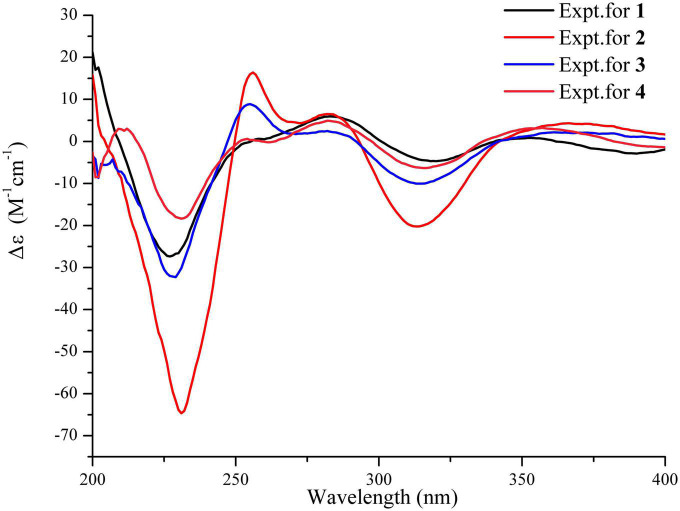
Comparisons of experimental CD (MeOH) spectra between compounds 1–4.

Correlations between OH-9 and H-18/OH-8/H-12, H-8, and H-19, and N1-H and H-7/H-18 in the NOESY experiment (Figure 3) suggested that the relative configurations of C-8, C-9, and C-12 were the same as those of 4. The absolute configurations of 1 were finally confirmed to be 2*S*, 8*S*, 9*R*, 12*S*, 18*S* by CD spectrum, which showed almost identical cotton effect curves compared to that of 4, demonstrating positive cotton effect at 283/353 nm and negative cotton effect at 227/320/390 nm (Figure 4).

Compound 2 was isolated as an amorphous yellow powder, and the molecular formula was assigned as C_22_H_24_N_3_O_7_ by HRESIMS (*m/z* 442.1615, [M − H]^–^, calcd. 442.1620), requiring 12 degrees of unsaturation. The ^1^H NMR data of 2 showed signals of three aromatic protons at δ_*H*_ 6.28 (dd, *J* = 8.6, 2.2, H-5), 6.46 (d, *J* = 2.2, H-7), and 7.27 (d, *J* = 8.6, H-4), one methoxy group (δ_*H*_ 3.80), and two methyl groups (δ_*H*_ 1.30, 1.58). The ^13^C and DEPT135 NMR spectra showed 22 carbons, including three carbonyl carbons (δ_*C*_ 195.3, 167.0, 165.6), four sp^2^ quaternary carbons (δ_*C*_ 167.1, 163.8, 137.7, 113.3), four sp^2^ methines (δ_*C*_ 124.9, 120.9, 107.5, 94.6), three sp^3^ quaternary carbons (δ_*C*_ 88.8, 85.1, 75.3), two sp^3^ methines (δ_*C*_ 74.4, 61.0), three sp^3^ methylenes (δ_*C*_ 44.5, 35.8, 20.2), one methoxyl group (δ_*C*_ 55.4), and two methyl groups (δ_*C*_ 25.4, 18.0). The ^1^H NMR and ^13^C NMR data showed similarities with those of spirotryprostatin G (5), indicating a similar planner structure (Table 1). Furthermore, the value of specific optical rotation [α]${}_{D}^{25}$ − 69.1 (*c* 0.08, MeOH) for 2 was negative which was in agreement with that of 1 ([α]${}_{D}^{25}$ − 81.2), and the experimental CD spectrum of 2 also showed similar cotton effect as that of 1. The above-mentioned evidence strongly supported the absolute configurations of 2 as 2*S*, 8*S*, 9*R*, 12*R*, 18*S*.

**FIGURE 5:**
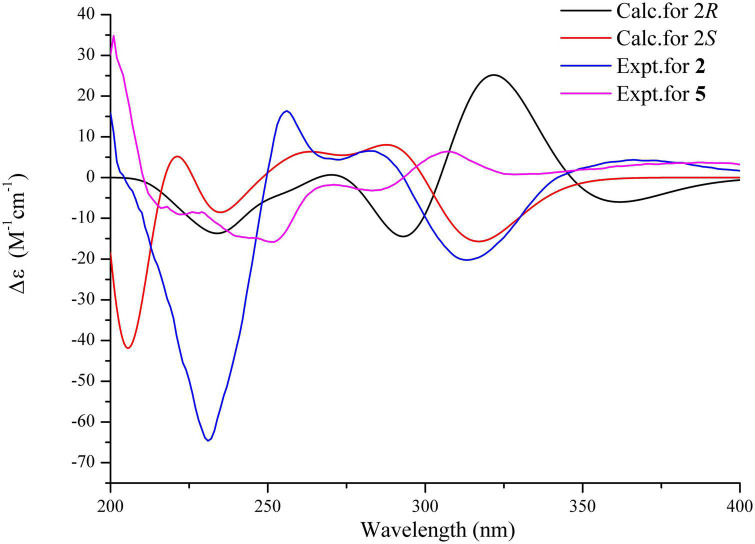
Experimental CD spectra of compounds 2 and 5 (MeOH) and ECD calculations of 2*R* and 2*S* configurations.

**FIGURE 6:**
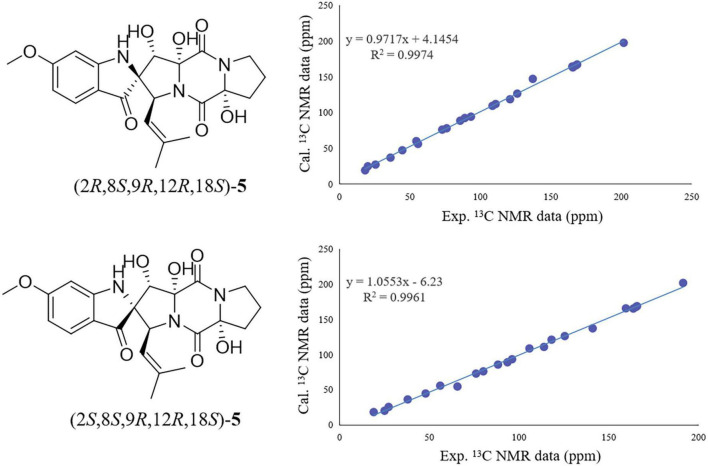
Regression analysis of experimental vs. calculated ^13^C NMR chemical shifts of 5 with 2*R*, 8*S*, 9*R*, 12*R*, 18*S* and 2*R*, 8*S*, 9*R*, 12*R*, 18*S* configurations at the mPW1PW91/6-31 + G(d,p) level.

On the contrary, the value of specific optical rotation for spirotryprostatin G (5) was positive ([α]${}_{D}^{25}$ + 60.9), which is opposite to that of 1, 2, and 4, indicating the differences of absolute configurations. Also, further analysis of NOESY correlations of 5 showed the key correlations between N1-H and H-19 and between H-8 and H-7/N1-H/H-19, suggesting that H-7/N1-H/H-8/H-19 were on the same side. The above-mentioned data illustrated that the configuration of spiro carbon at the C-2 position of 5 might be different from that of 1, 2, and 4. The experimental and computational calculation CD spectra of 5 were then applied to elucidate the absolute configurations. It showed that the experimental CD spectrum of 5 was quite different from that of 2. ECD calculations of 2*S* and 2*R* configurations of 5 were also applied consequently comparing with experimental CD spectra. It showed that the calculated ECD spectrum of 2*R* matched well with the experimental ECD spectrum of 5, while the calculated ECD spectrum of 2*S* matched well with the experimental ECD spectrum of 2 (Figure 5). Moreover, the ^13^C NMR chemical shifts of proposed structures for compound 5 with 2*R*, 8*S*, 9*R*, 12*R*, 18*S* and 2*S*, 8*S*, 9*R*, 12*R*, 18*S* configurations were subjected to calculate at the level of MPW1PW91/6-31G(d) with the PCM solvent model for DMSO. As a result, the calculated NMR values of (2*R*, 8*S*, 9*R*, 12*R*, 18*S*) of compound 5 was predicted to be the correct one with a DP4 + probability of 100% (using both H and C data) *via* comparing the data of candidate and experimental structures (Figure 6 and Supplementary Table S1). In addition, the values of the higher linear correlation coefficients (*R*^2^), the lower RMSD, MAD, and CMAD also support the assigned absolute configuration as 2*R*, 8*S*, 9*R*, 12*R*, 18*S* (Supplementary Table S2). Thus, the absolute configurations of 2 and 5 (spirotryprostatin G) were finally confirmed to be 2*S*, 8*S*, 9*R*, 12*R*, 18*S*, and 2*R*, 8*S*, 9*R*, 12*R*, 18*S*, respectively (Supplementary Tables S3–S8).

Compound 3, a pale yellow powder, exhibited the molecular formula C_22_H_25_N_3_O_6_, as determined from the HRESIMS (*m/z* 426.1671, [M − H]^–^, calcd. 426.1671), requiring 12 degrees of unsaturation. The ^1^H NMR spectrum showed three aromatic protons at δ_*H*_ 7.26 (d, *J* = 8.6, H-4), 6.46 (d, *J* = 2.1, H-7), and 6.28 (dd, *J* = 8.6, 2.2, H-5), one methoxy group (δ_*H*_ 3.79), and two methyl groups (δ_*H*_1.38, 1.57). The ^13^C NMR and DEPT135 NMR spectra showed signals for 22 carbons, including three carbonyl carbons (δ_*C*_ 195.3, 168.6, 164.8), four sp^2^ quaternary carbons (δ_*C*_ 166.09, 163.9, 133.6, 113.4), four sp^2^ methines (δ_*C*_ 124.8, 121.1, 107.5, 94.7), two sp^3^ quaternary carbons (δ_*C*_ 85.4, 75.5), three sp^3^ methine (δ_*C*_ 74.1, 60.7, 59.7), three sp^3^ methylene (δ_*C*_ 44.5, 27.8, 22.6), and three methyl groups (δ_*C*_ 55.4, 25.4, 17.9). The ^1^H NMR and ^13^C NMR data of 3 showed similarity to those of 2 and differed only in the absence of the hydroxyl group of 2 (Table 1). The planner structure of 3 was further determined by HSQC, COSY, and HMBC correlations. In the NOESY spectrum of 3, the obvious correlation signals between N-H and H-7/H-18, H-8 and H_2_-19, 8-OH, and H-12/9-OH were observed, indicating that these protons of H-7/N-H/8-OH/9-OH/H-12 were on the same side. Thus, the relative stereochemistry of 3 was determined. Further study showed that the specific optical rotation [α]${}_{D}^{25}$ − 151.1 (*c* 0.08, MeOH) for 3 was consistent with those of compounds 1, 2, and 4, which was opposite compared to that of the reported known compound spirotryprostatin C [α]${}_{D}^{25}$ + 147.2 (*c* 0.10, MeOH). The experimental ECD spectrum was then applied to determine the absolute configuration of 3. It showed that the experimental ECD spectrum of 3 had a similar Cotton effect curve with those of 2, suggesting 2*S* configurations, while the absolute configuration of spirotryprostatin C should be revised to 2*R* (Figure 4).

### Bioassay

All the compounds were evaluated for their antimicrobial activities toward *Pseudomonas aeruginosa* PAO1, *Dickeya zeae* EC1, *Staphylococcus epidermidis*, *Escherichia coli*, and *Sporisorium scitamineum*. Compound 7 displayed moderate inhibitory activity toward dimorphic switch of pathogenic smut fungi *Sporisorium scitamineum* at 25 μM. Compounds 3 and 6 showed weak antibacterial activities against phytopathogenic bacterial *Dickeya zeae* EC1 at 100 μM.

## Conclusion

In this study, we described that three new spirooxindole diketone piperazine derivatives, named spirobrefeldins A–C (1–3), together with four known indole diketone piperazine analogs were isolated from *Penicillium brefeldianum*. The absolute configurations of compounds 1–5 were determined by CD spectra together with ECD calculations. The absolute configurations of C-2 chiral carbon in spirotryprostatin G (5) and spirotryprostatin C were revised accordingly. After preliminary antimicrobial inhibitory bioassays of them, compound 7 displayed moderate inhibitory activity toward the dimorphic switch of pathogenic smut fungi *Sporisorium scitamineum* at 25 μM. Compounds 3 and 6 showed weak antibacterial activities against phytopathogenic bacterial *Dickeya zeae* EC1 at 100 μM.

## Data availability statement

The original contributions presented in this study are included in the article/Supplementary material, further inquiries can be directed to the corresponding author.

## Author contributions

HS and JJ did the experiments. JJ wrote the draft. HZ calculated the ECD spectra and determined the absolute structures. HJ measured and analyzed the NMR data. ZS did the fermentation and got crude extract. DL purified the strain from soil samples. LJ gave some advices on writing. FH designed the experiment, got the fundings, and wrote the manuscript. All authors contributed to the article and approved the submitted version.
