# Supplementary material for: Antibacterial spirooxindole alkaloids from Penicillium brefeldianum inhibit dimorphism of pathogenic smut fungi
Source: Front Microbiol. 2022 Nov 14;13:1046099. doi: 10.3389/fmicb.2022.1046099 (PMC9702524; doi:10.3389/fmicb.2022.1046099)
Supplement: Supplementary file 1 [file Data_Sheet_1.pdf]

Supporting Information

Antibacterial Spirooxindole Alkaloids from *Penicillium brefeldianum* Inhibit Dimorphism of Pathogenic Smut Fungi

Huajun Shi[1,#], Jinyan Jiang[1,2#], Hang Zhang[1,#], Haimei Jiang[1], Zijie Su[1], Dandan Liu[3], Ligang Jie[1,3], Fei He[1,3*]

Table of content

No.	Content	Page
1	**Figure S1** ^{1}H NMR spectrum of spirobrefeldin A (**1**, 600 MHz, DMSO-d_6)	4
2	**Figure S2** ^{13}C NMR spectrum of spirobrefeldin A (**1**, 150 MHz, DMSO-d_6)	5
3	**Figure S3:** ^{1}H-^{1}H COSY spectrum of spirobrefeldin A (**1**, DMSO-d_6)	6
4	**Figure S4** HSQC spectrum of spirobrefeldin A (**1**, DMSO-d_6),	7
5	**Figure S5** HMBC spectrum of spirobrefeldin A (**1**, DMSO-d_6)	8
6	**Figure S6** NOESY spectrum of spirobrefeldin A (**1**, DMSO-d_6)	9
7	**Figure S7** HRESIMS spectrum of spirobrefeldin A (**1**)	10
8	**Figure S8** IR spectrum of spirobrefeldin A (**1**)	11
9	**Figure S9** CD spectrum of spirobrefeldin A (**1**)	12
10	**Figure S10** ^{1}H NMR spectrum of spirobrefeldin B (**2**, 600 MHz, DMSO-d_6)	13
11	**Figure S11** ^{13}C NMR spectrum of spirobrefeldin B (**2**, 150 MHz, DMSO-d_6)	14
12	**Figure S12** ^{1}H-^{1}H COSY spectrum of spirobrefeldin B (**2**, DMSO-d_6)	15
13	**Figure S13** HSQC spectrum of spirobrefeldin B (**2**, DMSO-d_6),	16
14	**Figure S14** HMBC spectrum of spirobrefeldin B (**2**, DMSO-d_6)	17
15	**Figure S15** NOESY spectrum of spirobrefeldin B (**2**, DMSO-d_6)	18
16	**Figure S16** HRESIMS spectrum of spirobrefeldin B (**2**)	19
17	**Figure S17** IR spectrum of spirobrefeldin B (**2**)	20
18	**Figure S18** CD spectrum of spirobrefeldin B (**2**)	21
19	**Figure S19** ^{1}H NMR spectrum of spirobrefeldin C (**3**, 600 MHz, DMSO-d_6)	22
20	**Figure S20** ^{13}C NMR spectrum of spirobrefeldin C (**3**, 150 MHz, DMSO-d_6)	23
21	**Figure S21** HSQC spectrum of spirobrefeldin C (**3**, DMSO-d_6)	24
22	**Figure S22** HMBC spectrum of spirobrefeldin C (**3**, DMSO-d_6)	25
23	**Figure S23** ^{1}H-^{1}H COSY spectrum of spirobrefeldin C (**3**, DMSO-d_6)	26
24	**Figure S24** NOESY spectrum of spirobrefeldin C (**3**, DMSO-d_6)	27
25	**Figure S25** HRESIMS spectrum of spirobrefeldin C (**3**)	28
26	**Figure S26** IR spectrum of spirobrefeldin C (**3**)	29
27	**Figure S27** CD spectrum of spirobrefeldin C (**3**)	30
36	**Figure S28** ^{1}H NMR spectrum of **4** (600 MHz, DMSO-d_6)	31
37	**Figure S29** ^{13}C NMR spectrum of **4** (150 MHz, DMSO-d_6)	32
38	**Figure S30** ^{1}H NMR spectrum of **5** (600 MHz, DMSO-d_6)	33
39	**Figure S31** ^{13}C NMR spectrum of **5** (150 MHz, DMSO-d_6)	34
40	**Figure S32** ^{1}H NMR spectrum of **6** (600 MHz, DMSO-d_6)	35
41	**Figure S33** ^{13}C NMR spectrum of **6** (150 MHz, DMSO-d_6)	36
42	**Figure S34** ^{1}H NMR spectrum of **7** (600 MHz, DMSO-d_6)	37
43	**Figure S35** ^{13}C NMR spectrum of **7** (150 MHz, DMSO-d_6)	38
44	**Figure S36** HSQC spectrum of spirotryprostatin G(**5**, DMSO-d_6)	39
45	**Figure S37** HMBC spectrum of spirotryprostatin G(**5**, DMSO-d_6)	40
46	**Figure S38** NOESY spectrum of spirotryprostatin G(**5**, DMSO-d_6)	41

47 **Table S1** DP4+ results of candidate configurations (2R,8S,9R,12R,18S)-5(Isomer 1) and (2S,8S,9R,12R,18S)-5(Isomer 2) 42

48 **Table S2** Linear correlation coefficients (R2), root-mean-square deviation (RMSD) mean absolute deviation (MAD) and corrected mean absolute deviation (CMAD) analyses of the calculated and experimental NMR data. 43

49 **Table S3** Energy analyses of conformers (2R,8S,9R,12R,18S)-5a-(2R,8S,9R,12R,18S)-5b 43

50 **Table S4** Experimental 13C-NMR chemical shifts and GIAO isotropic magnetic shielding values calculated for PCM/mPW1PW91/6-31+G (d, p) geometries of (2R,8S,9R,12R,18S)-5 44

51 **Table S5** Experimental 1H-NMR chemical shifts and GIAO isotropic magnetic shielding values calculated for PCM/mPW1PW91/6-31+G (d, p) geometries of (2R,8S,9R,12R,18S)-5 45

52 **Table S6** Energy analyses of conformers (2S,8S,9R,12R,18S)-5a-(2S,8S,9R,12R,18S)-5h 46

53 **Table S7** Experimental 13C-NMR chemical shifts and GIAO isotropic magnetic shielding values calculated for PCM/mPW1PW91/6-31+G (d, p) geometries of (2S,8S,9R,12R,18S)-5 47

54 **Table S8** Experimental 1H-NMR chemical shifts and GIAO isotropic magnetic shielding values calculated for PCM/mPW1PW91/6-31+G (d, p) geometries of (2S,8S,9R,12R,18S)-5 48

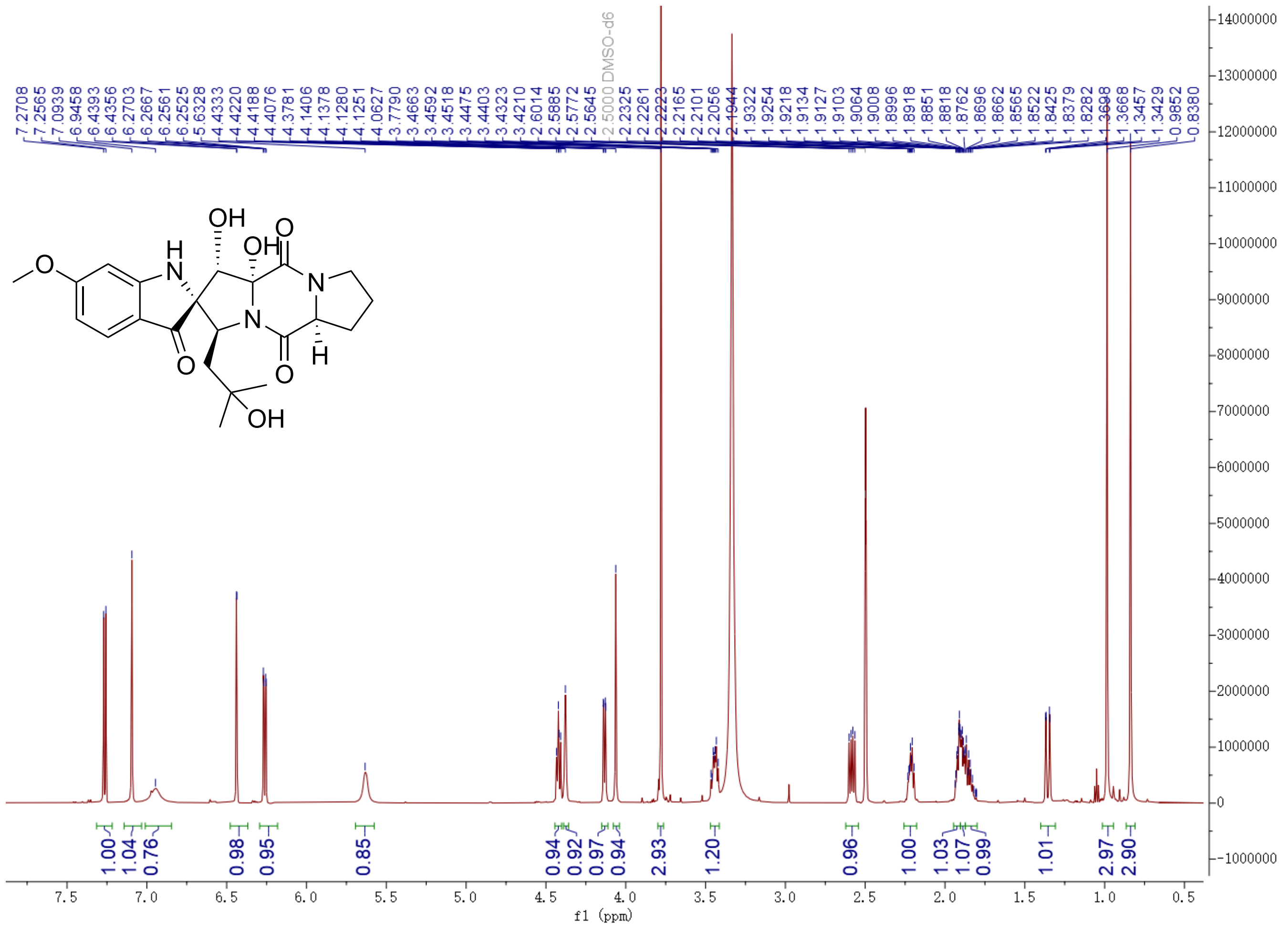

Fig. S1 ^{1}H NMR spectrum of **1**

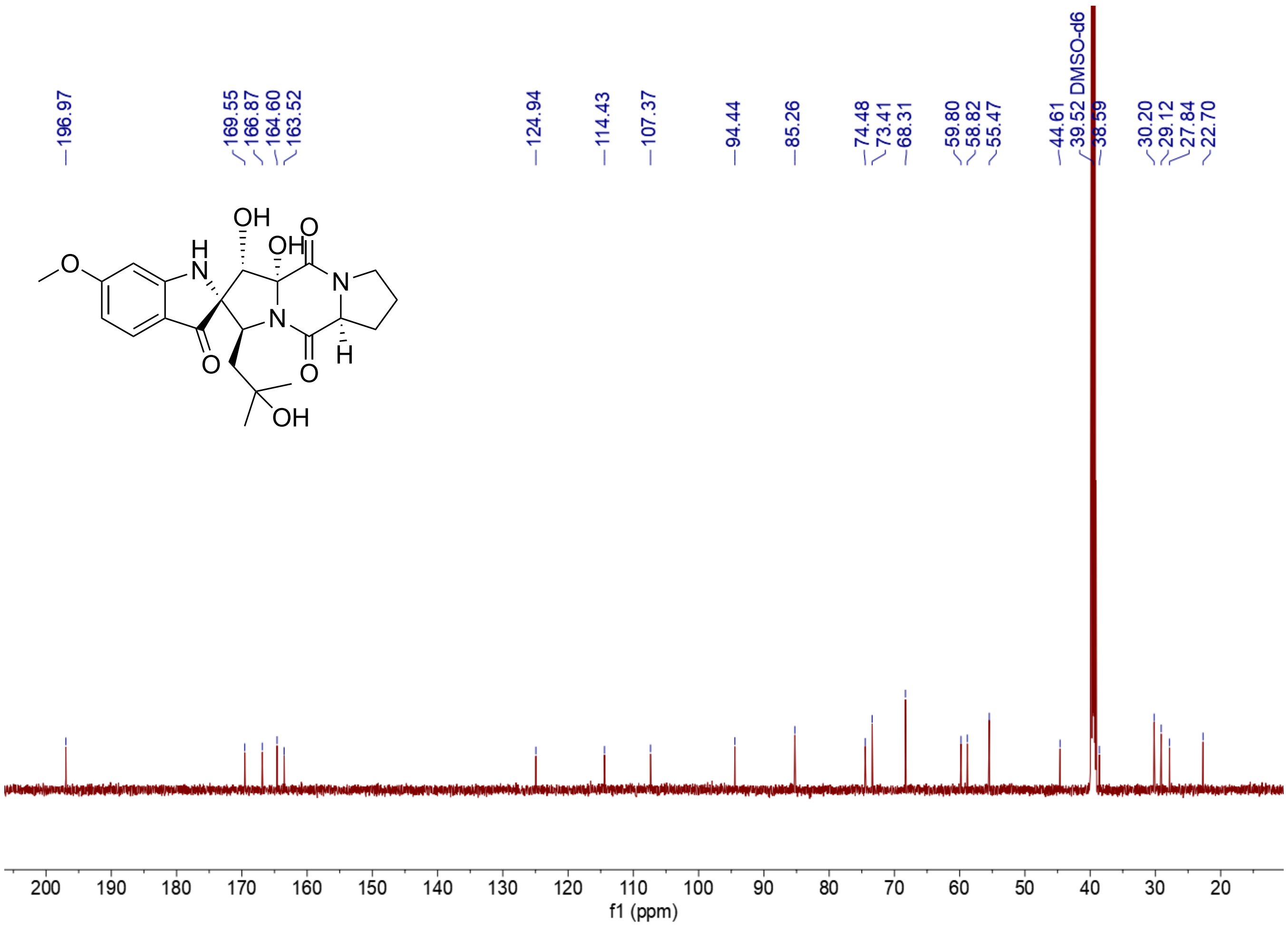

Fig. S2 ^{13}C NMR spectrum of **1**

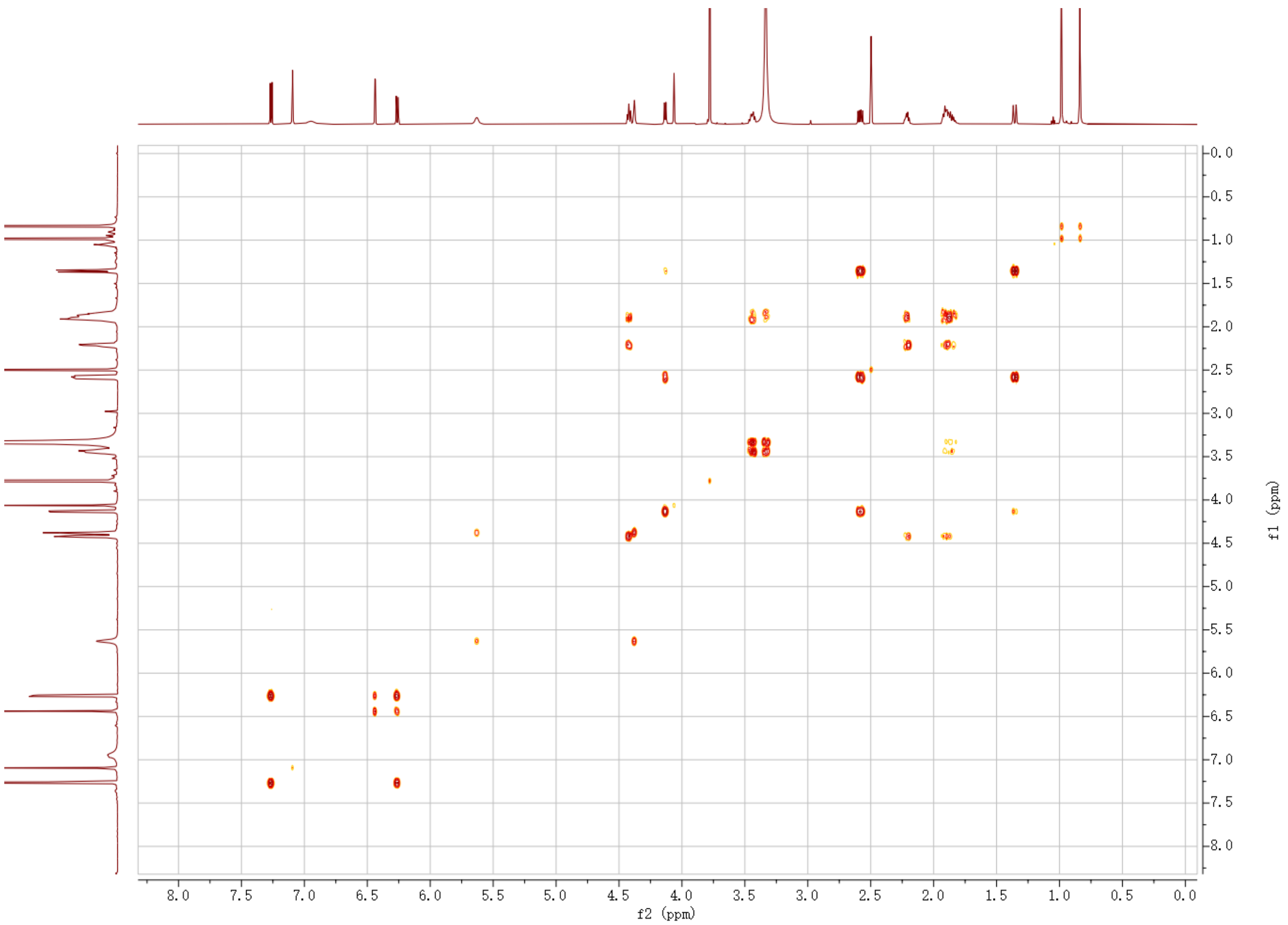

Fig. S3 ^1H-^1H COSY spectrum of **1**

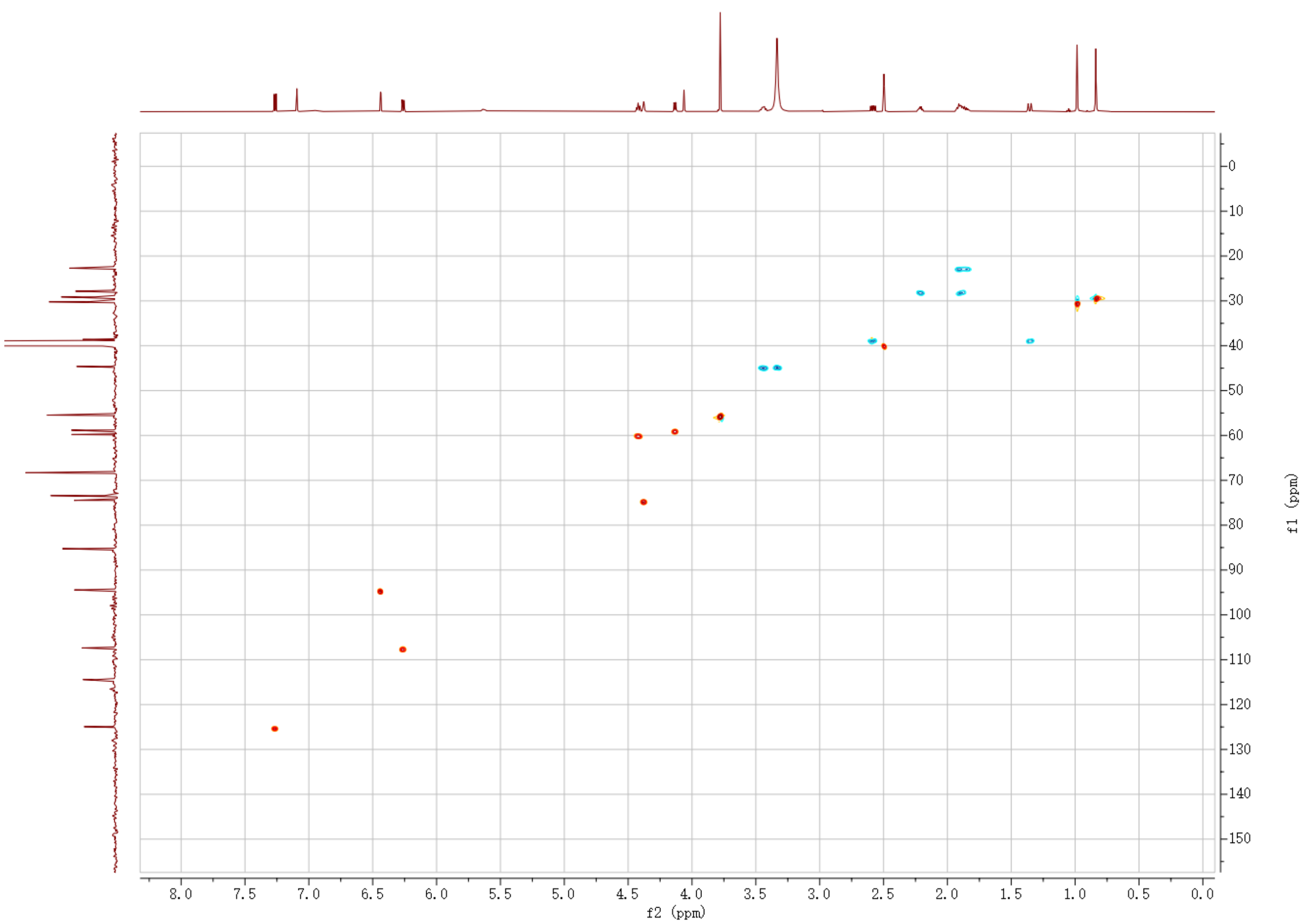

Fig. S4 HSQC spectrum of **1**

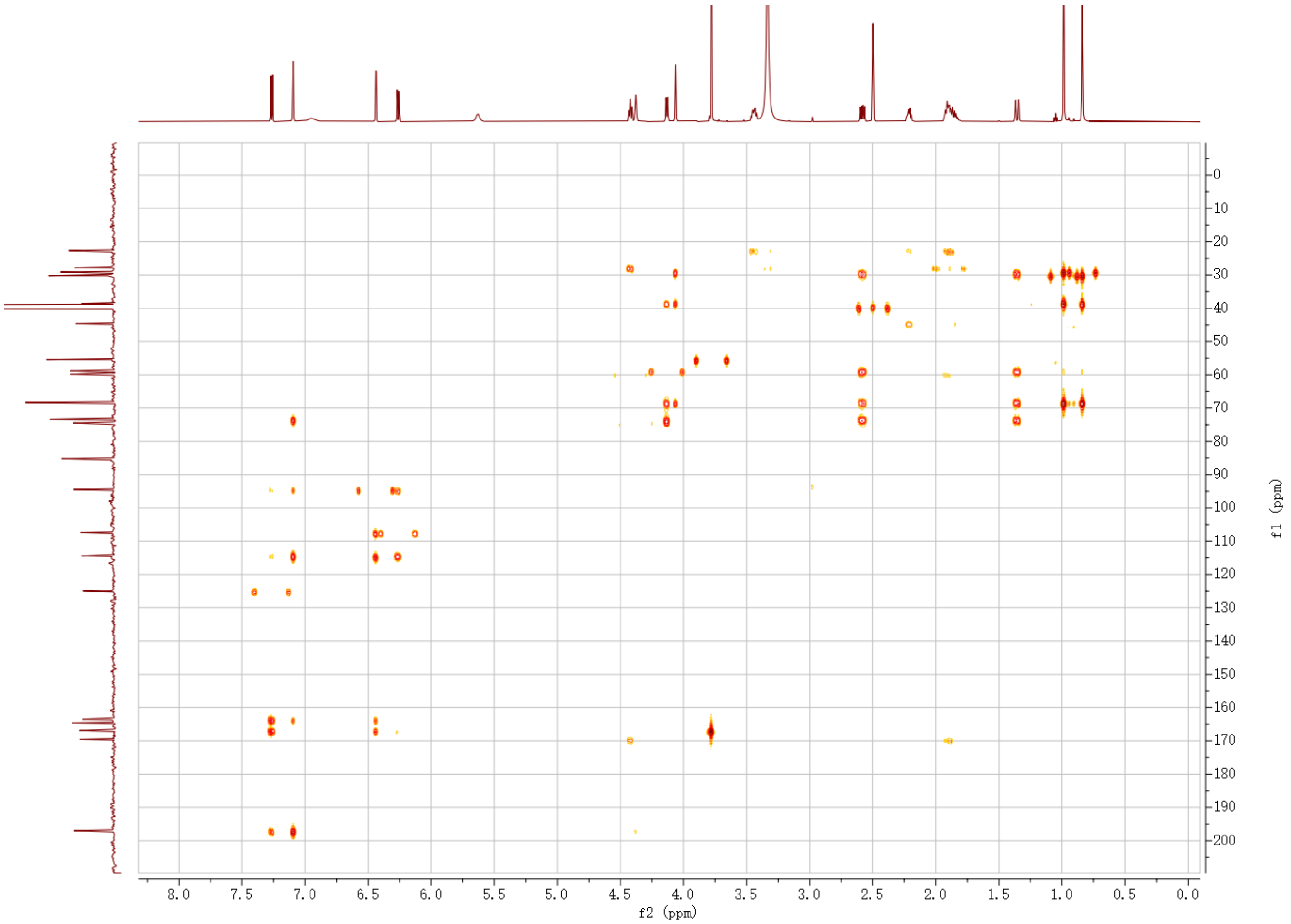

Fig. S5 HMBC spectrum of **1**

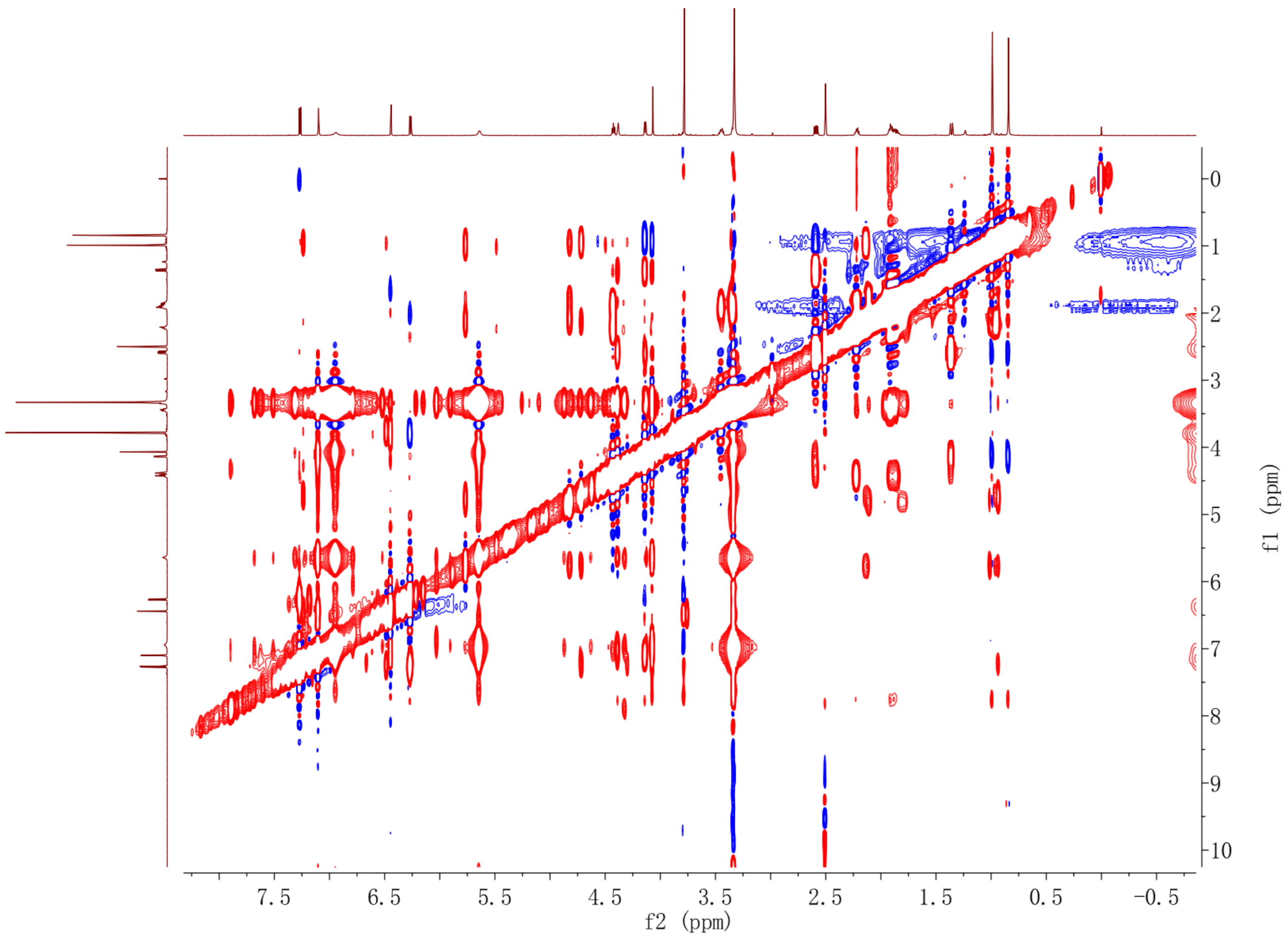

Fig. S6 NOESY spectrum (600 MHz, DMSO) of Compound **1**

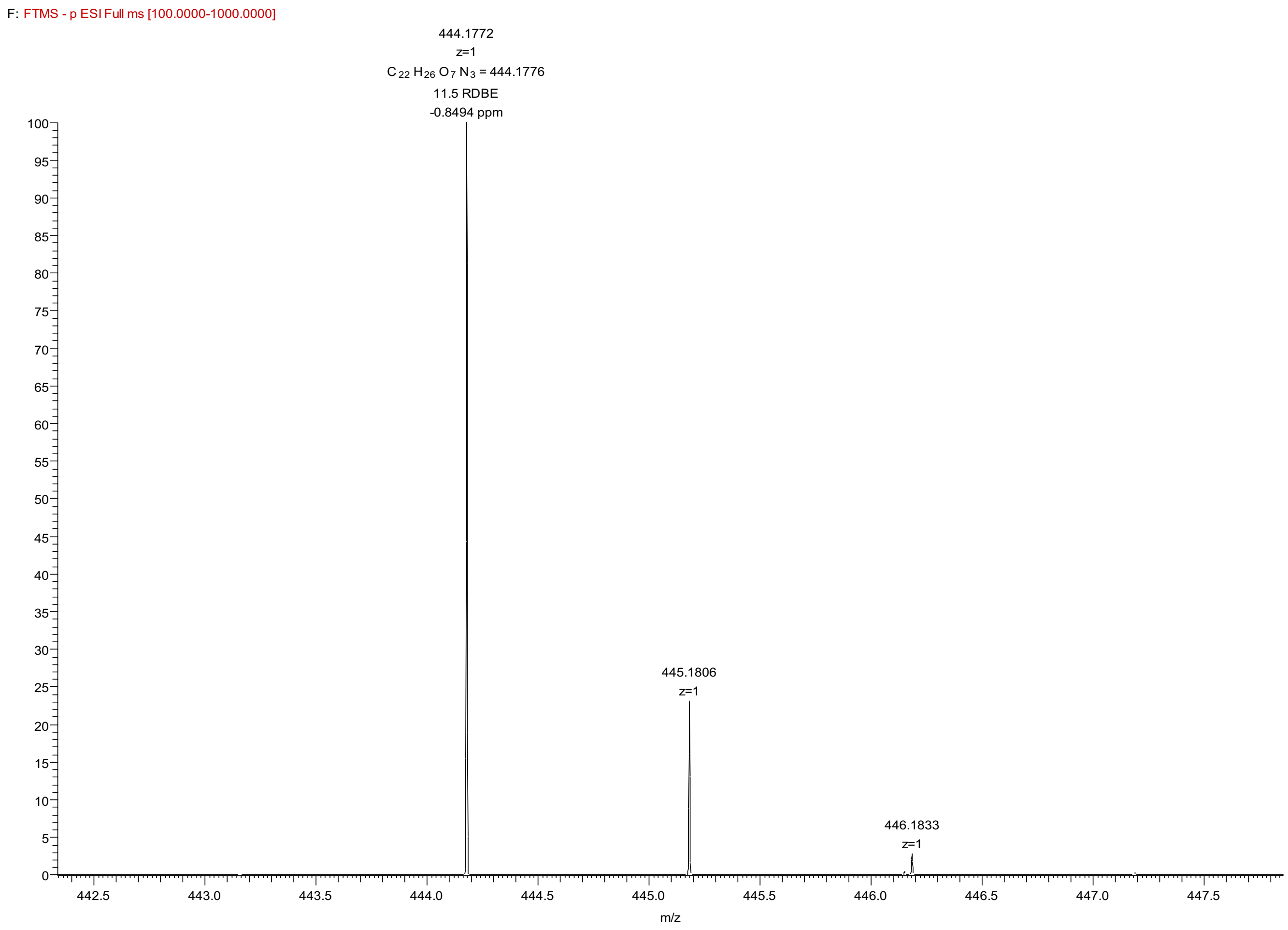

Fig. S7 HRESIMS spectrum of **1**

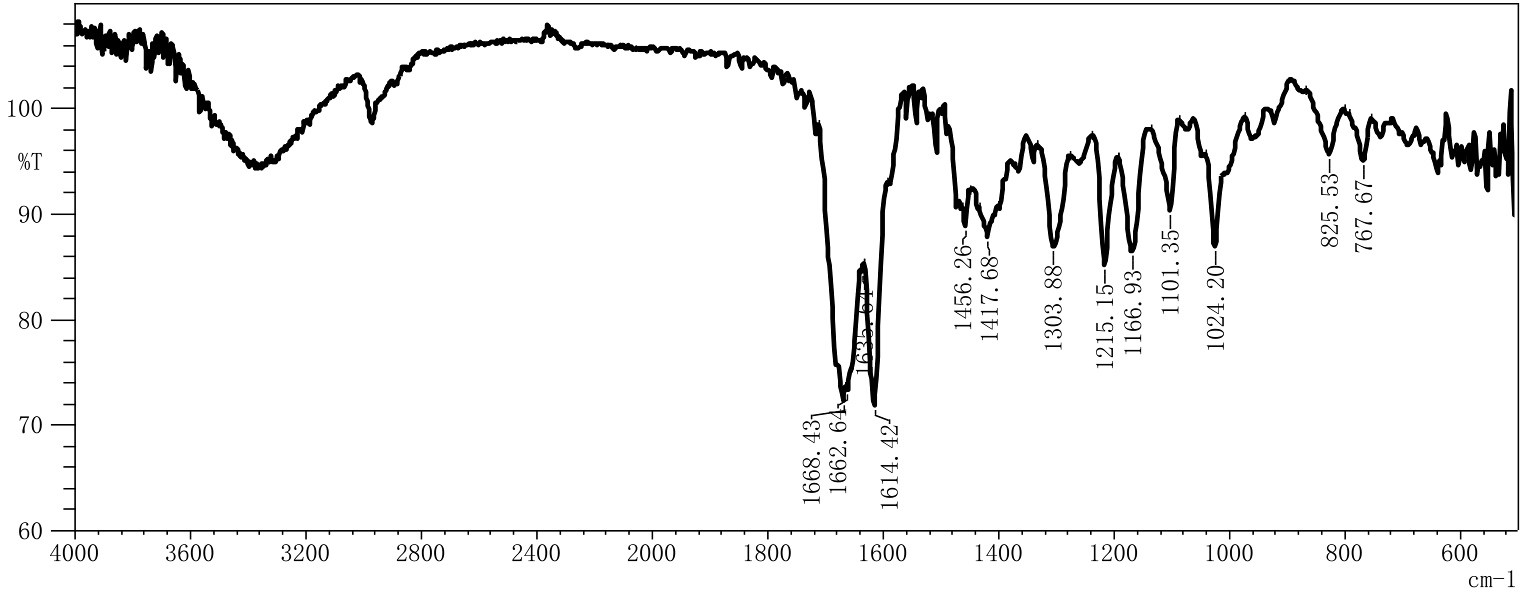

Fig. S8 IR spectrum of **1**

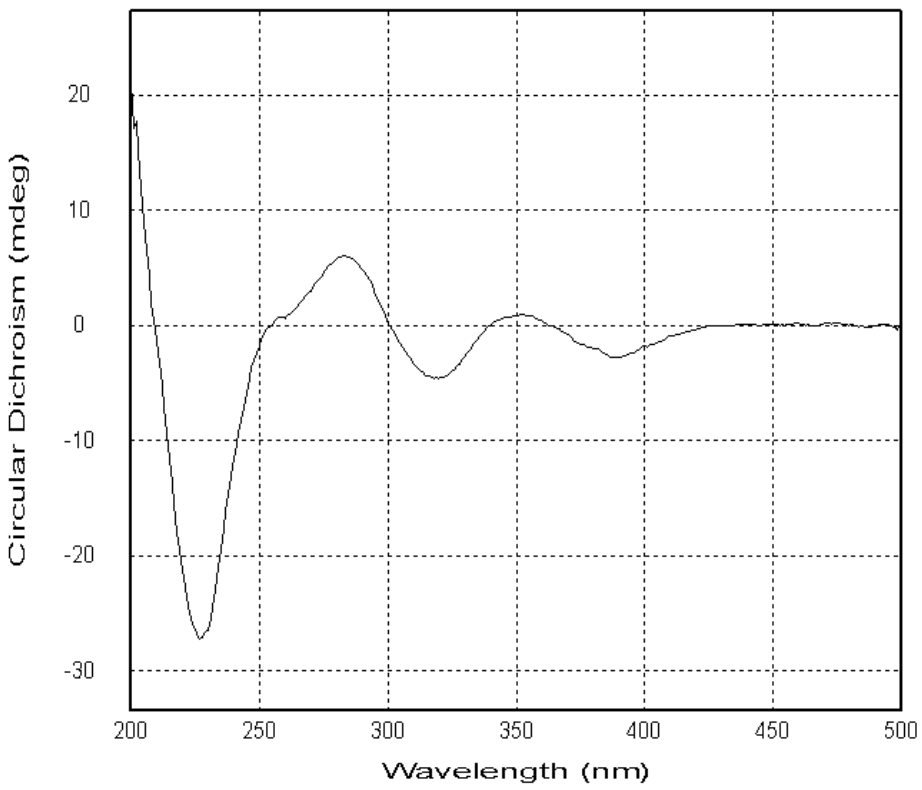

Fig. S9 Experimental CD spectrum of **1**

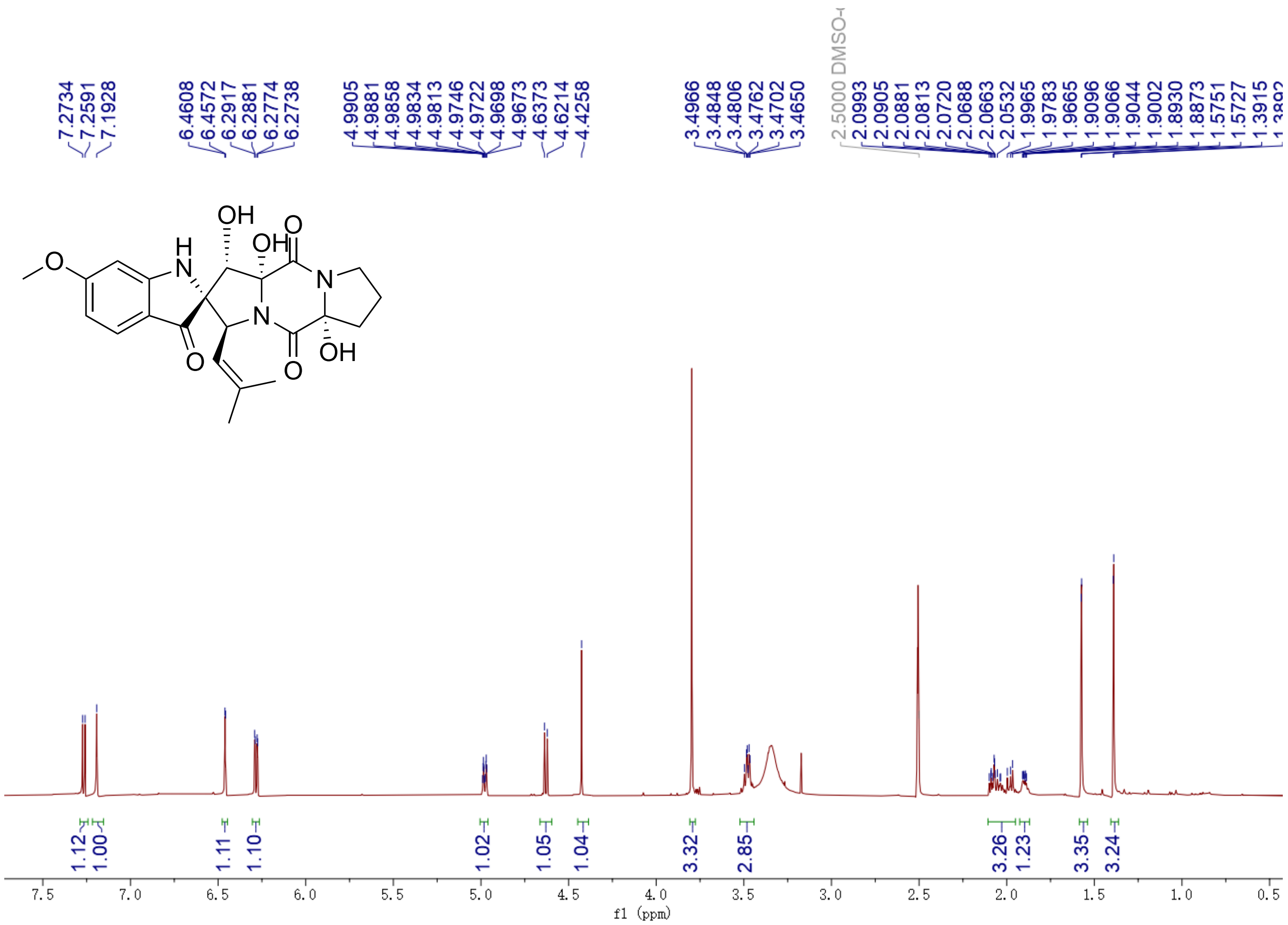

Fig. S10 ¹H NMR spectrum of **2**

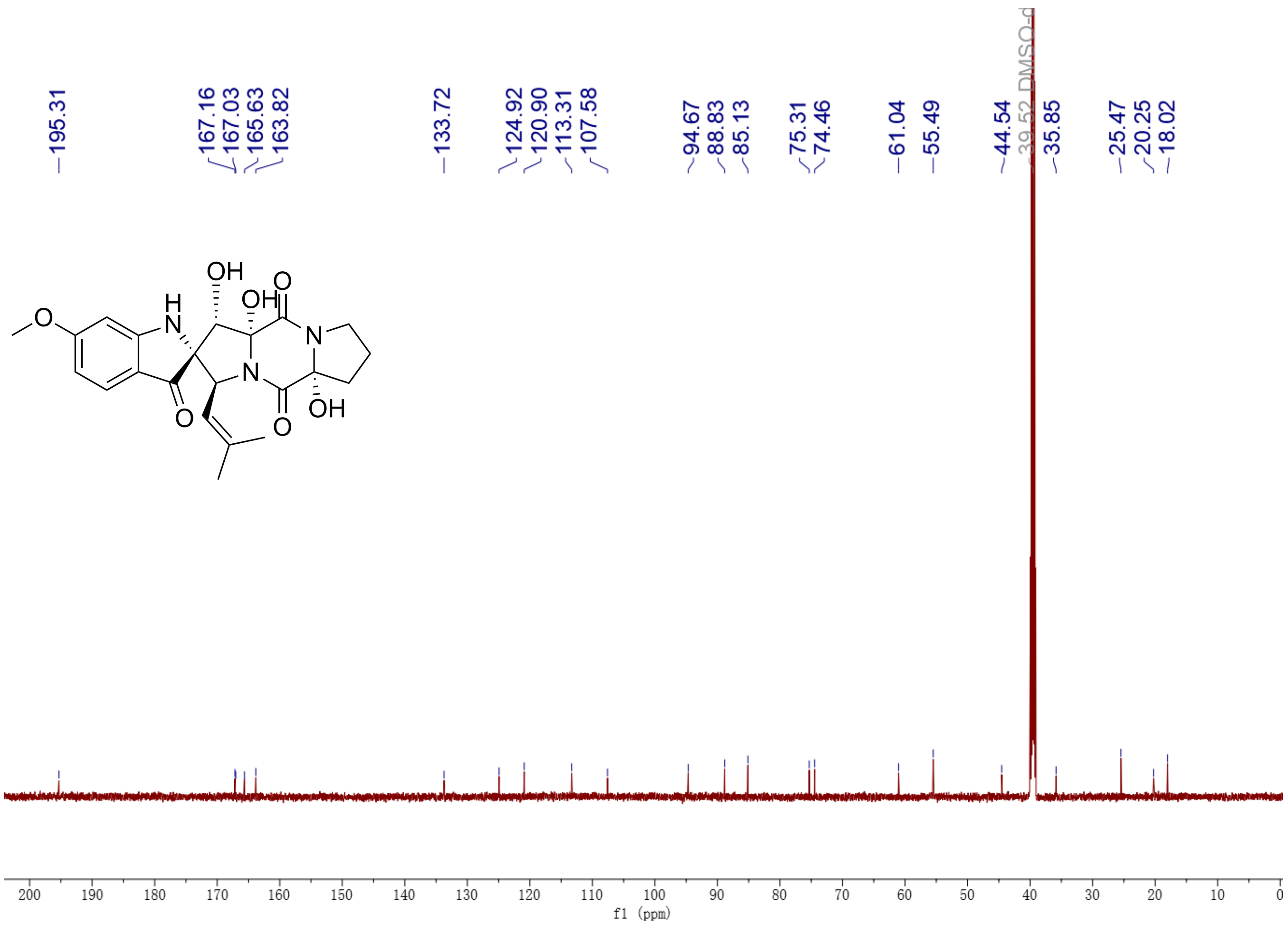

Fig. S11 ^{13}C NMR spectrum of **2**

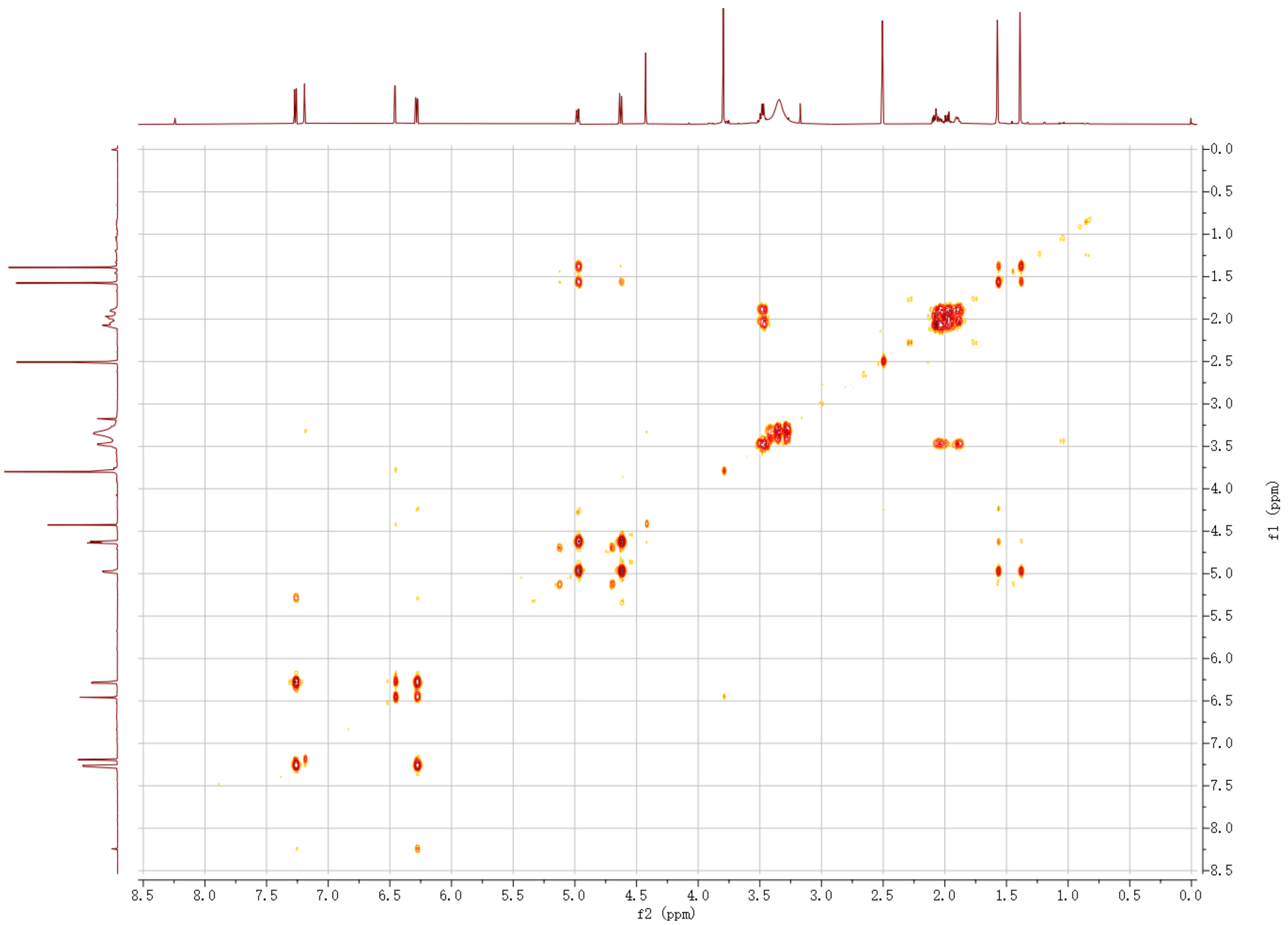

Fig. S12 ^{1}H-^{1}H COSY spectrum of **2**

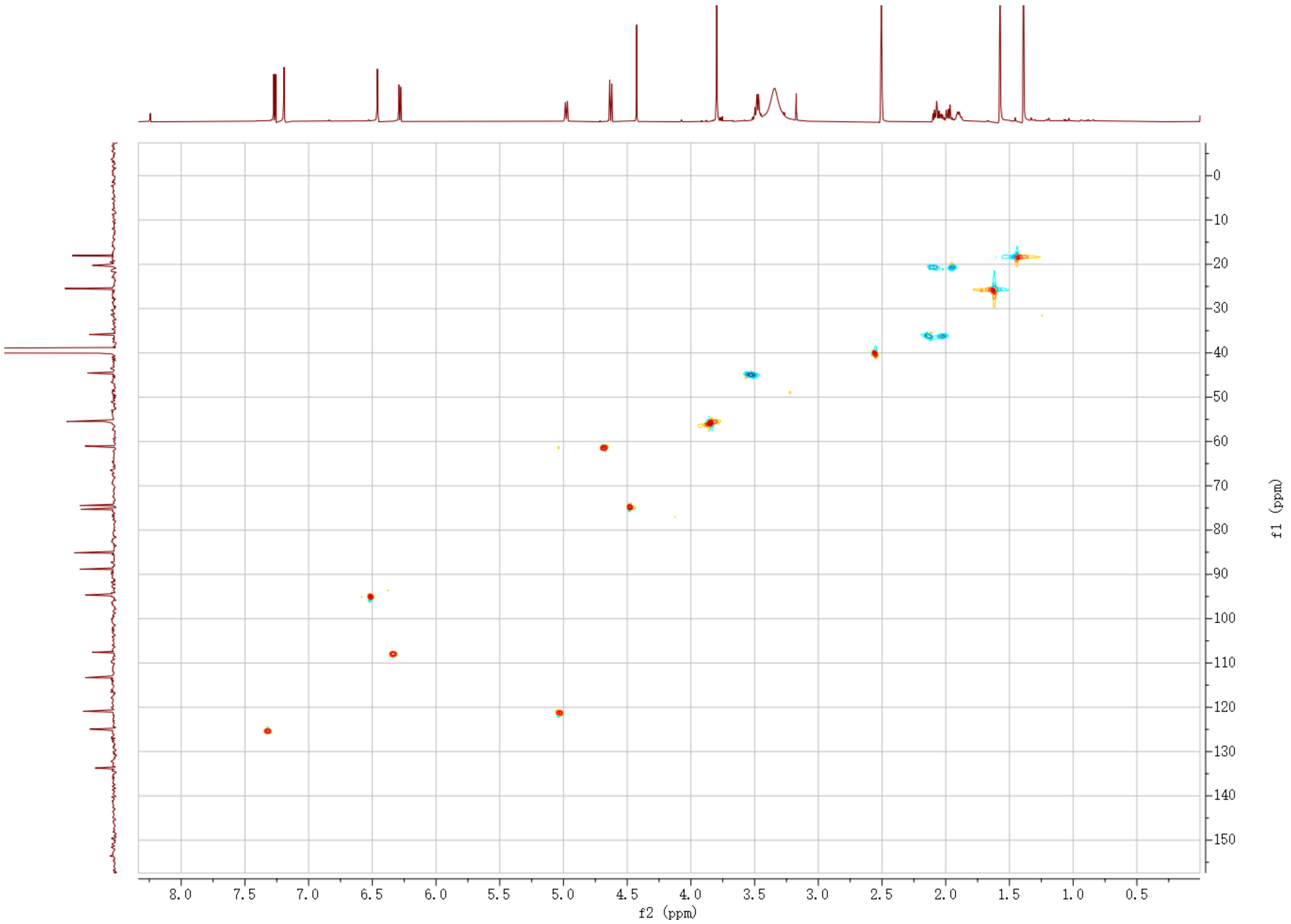

Fig. S13 HSQC spectrum of **2**

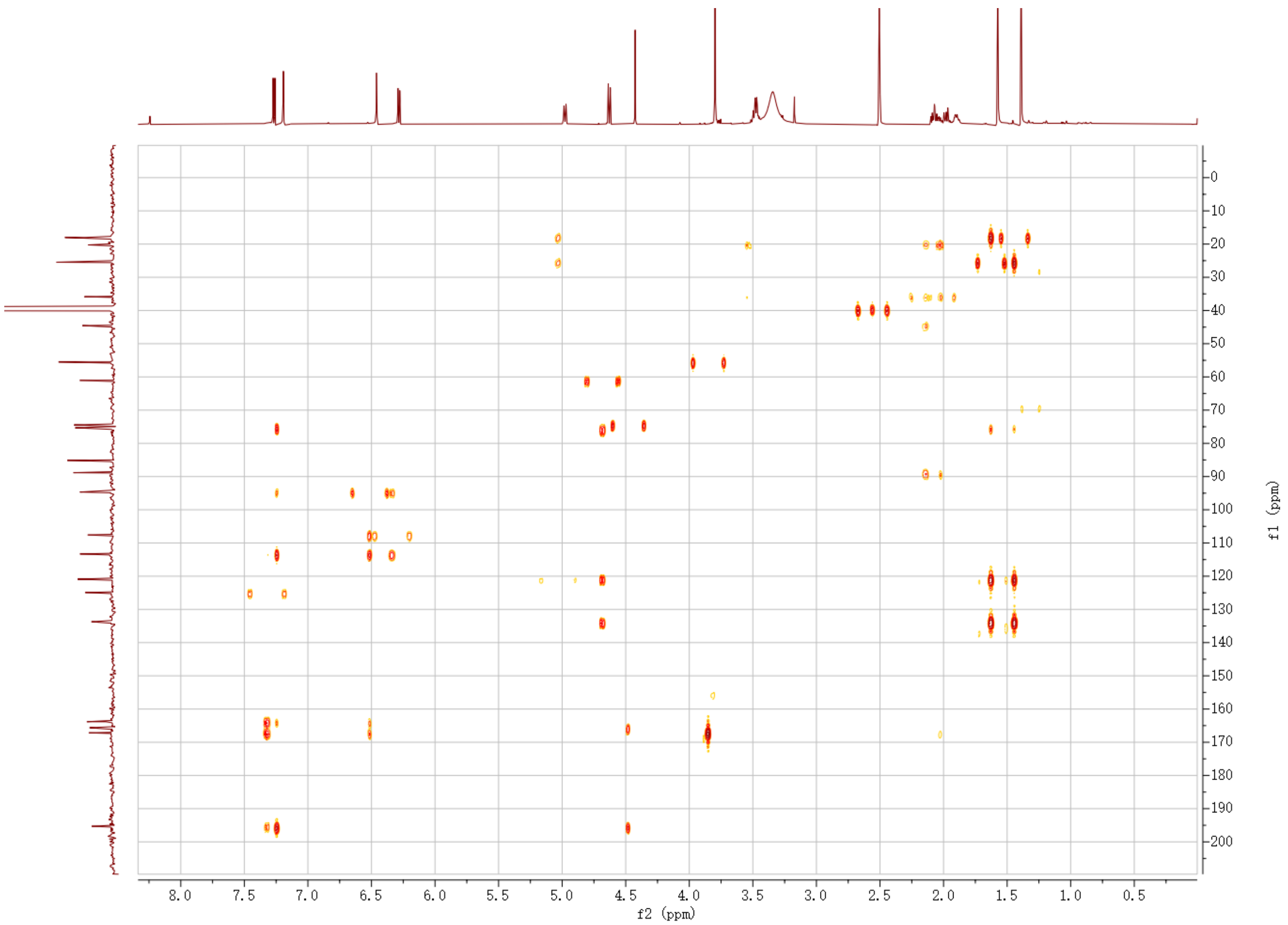

Fig. S14 HMBC spectrum of **2**

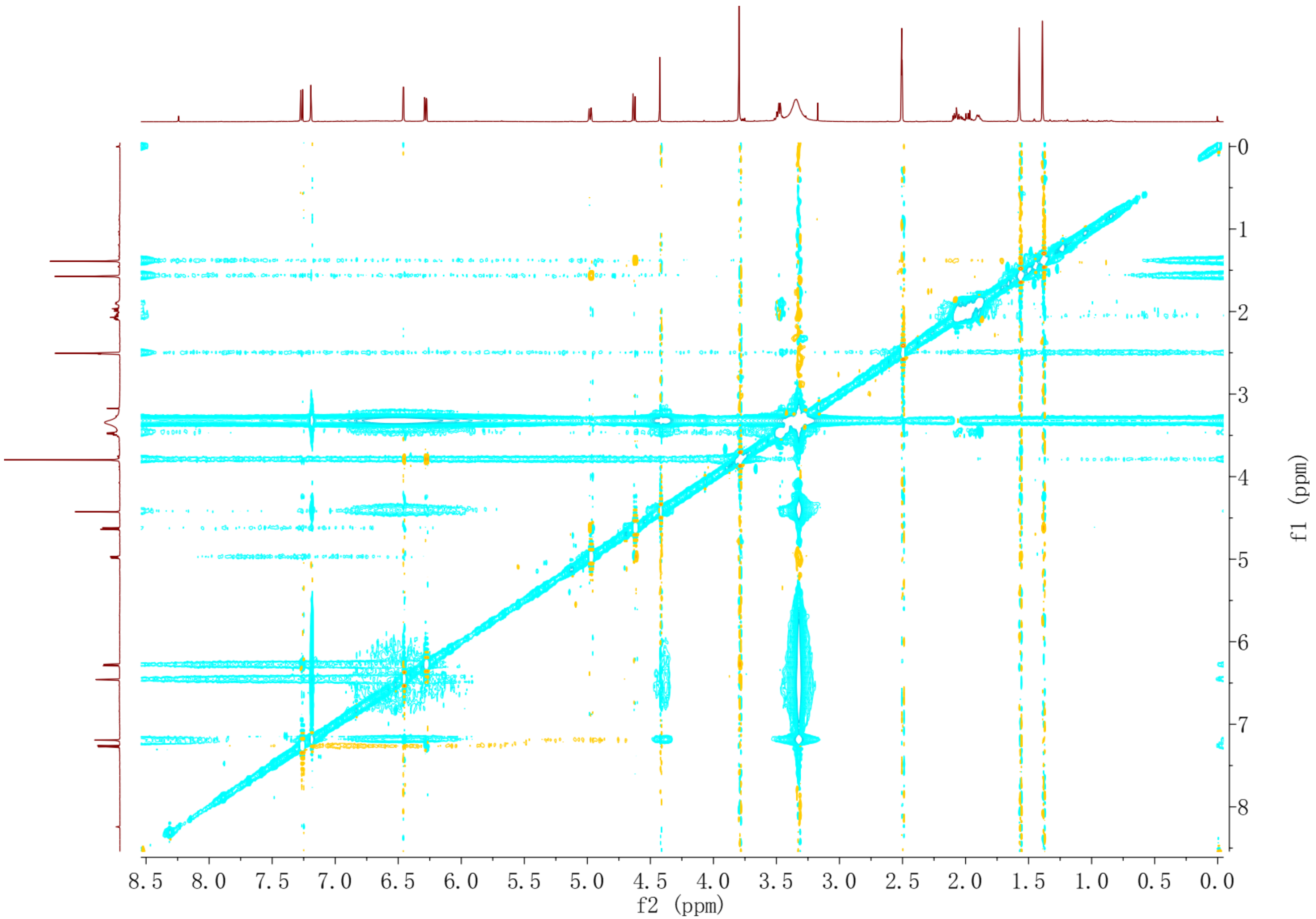

Fig. S15 NOESY spectrum of **2**

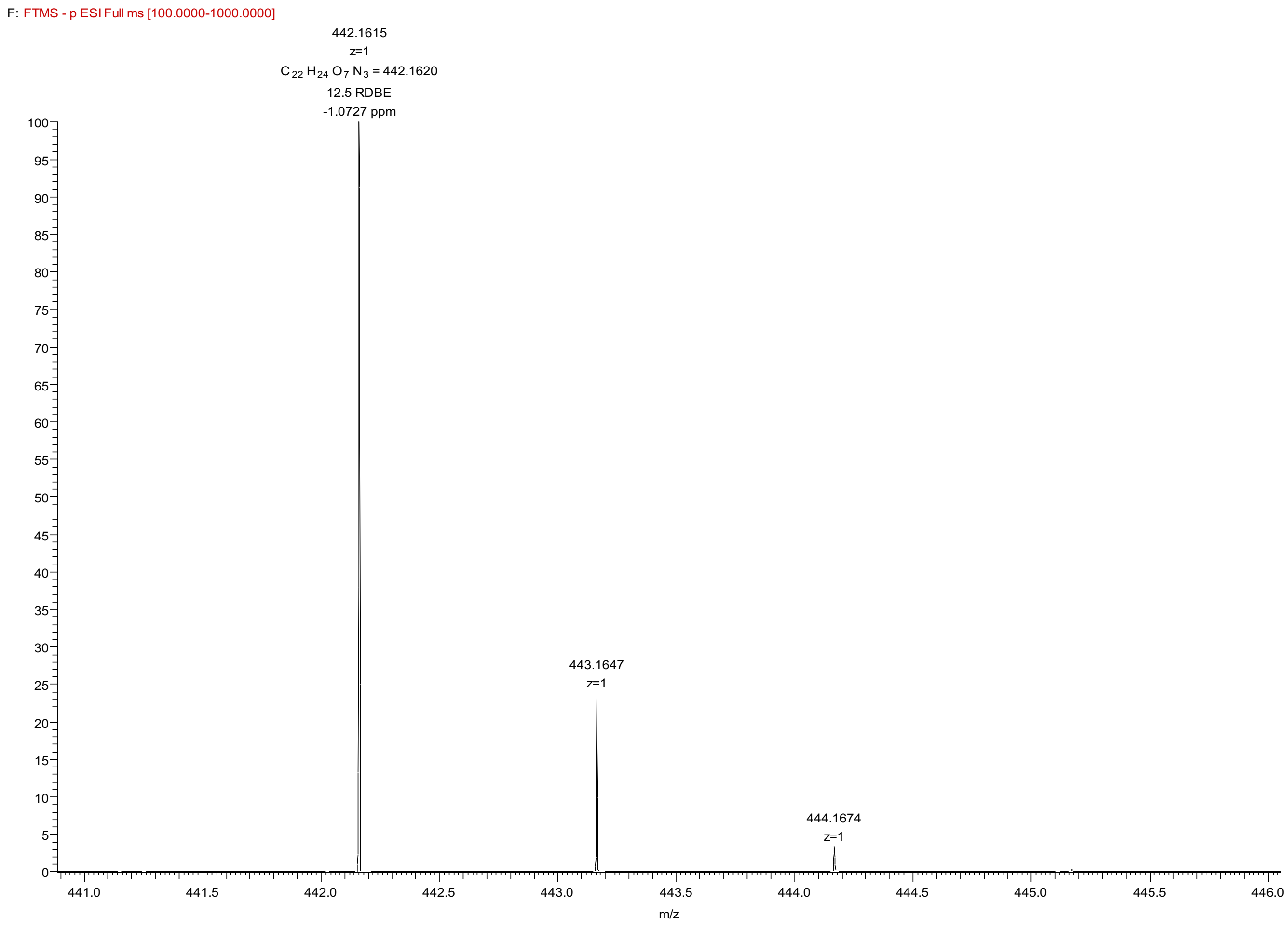

Fig. S16 HRESIMS spectrum of **2**

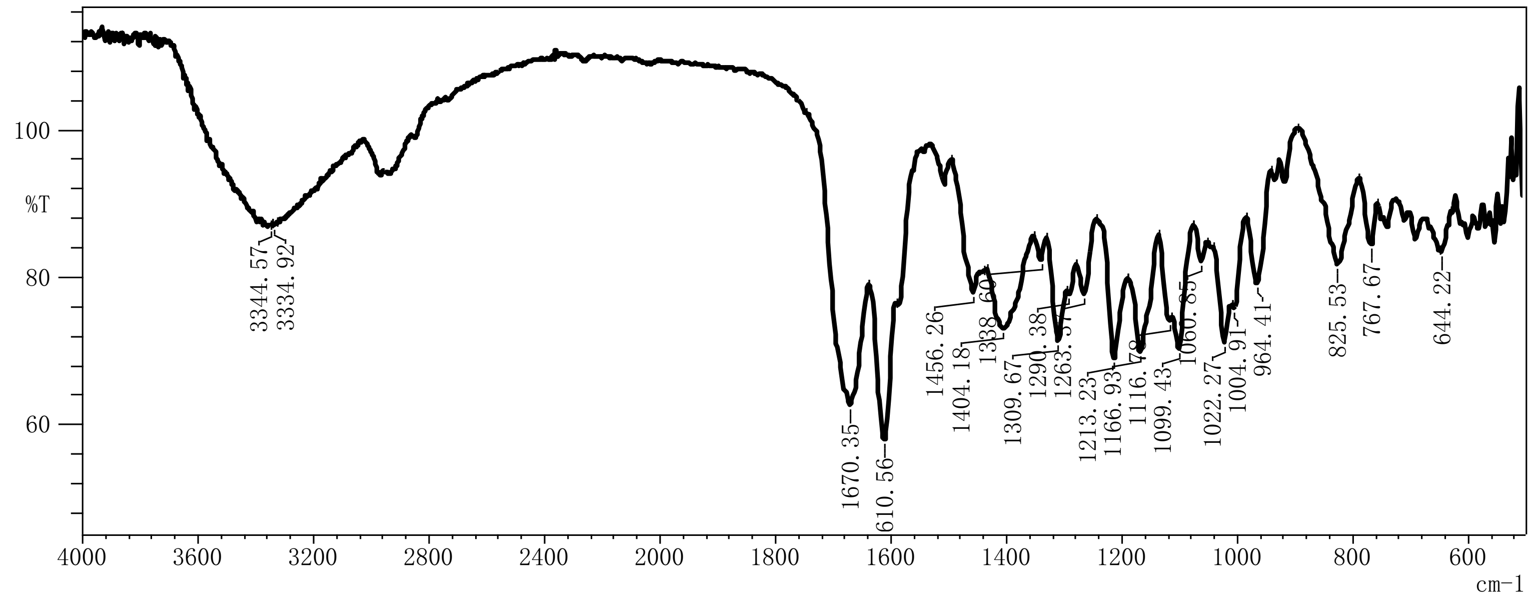

Fig. S17 IR spectrum of **2**

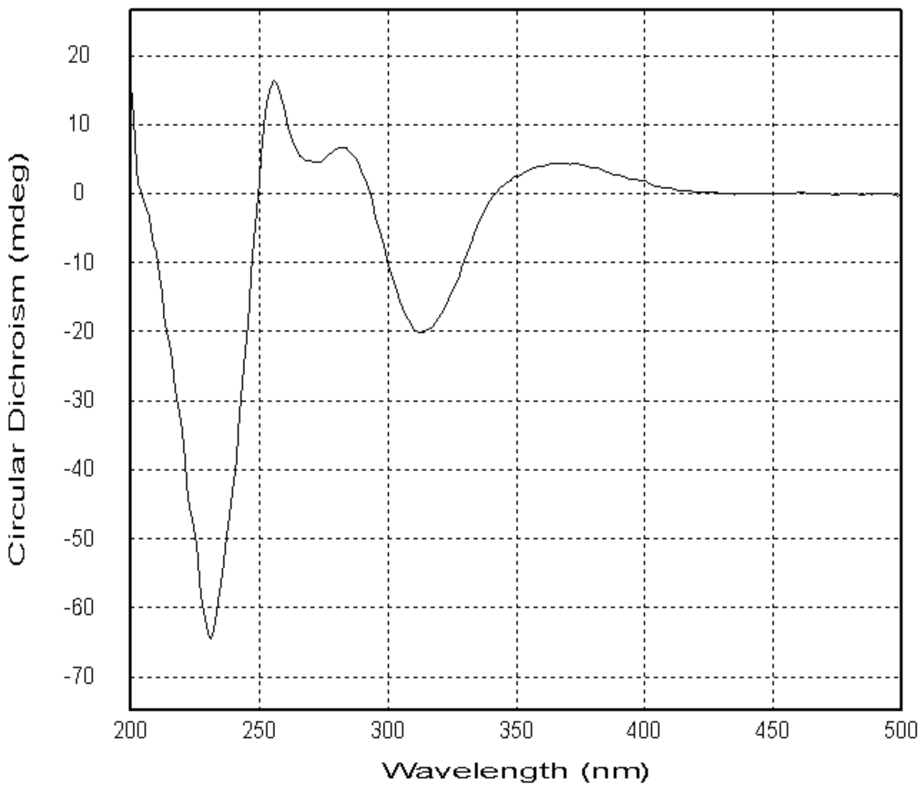

Fig. S18 Experimental CD spectrum of **2**

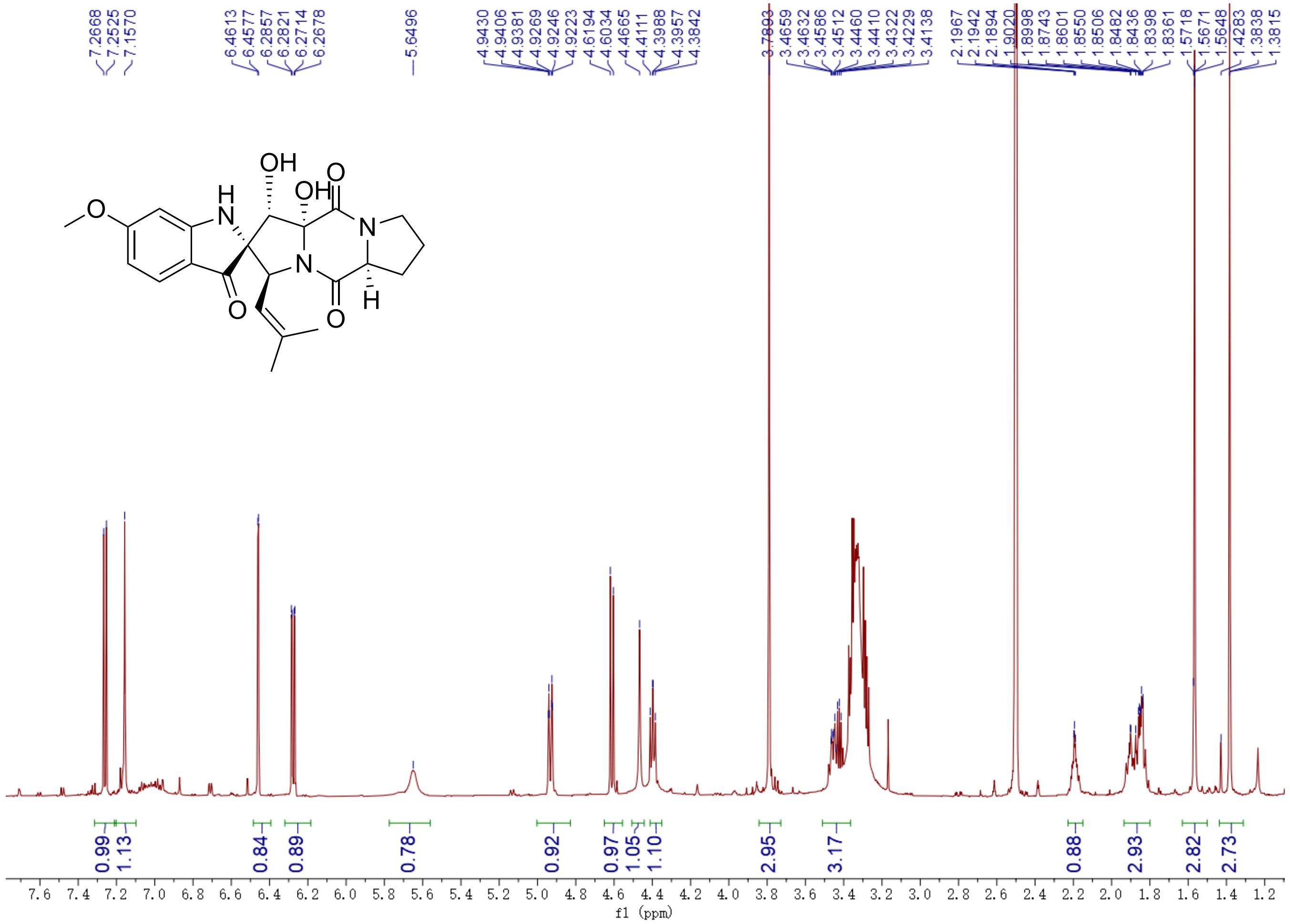

Fig. S19 ^{1}H NMR spectrum of **3**

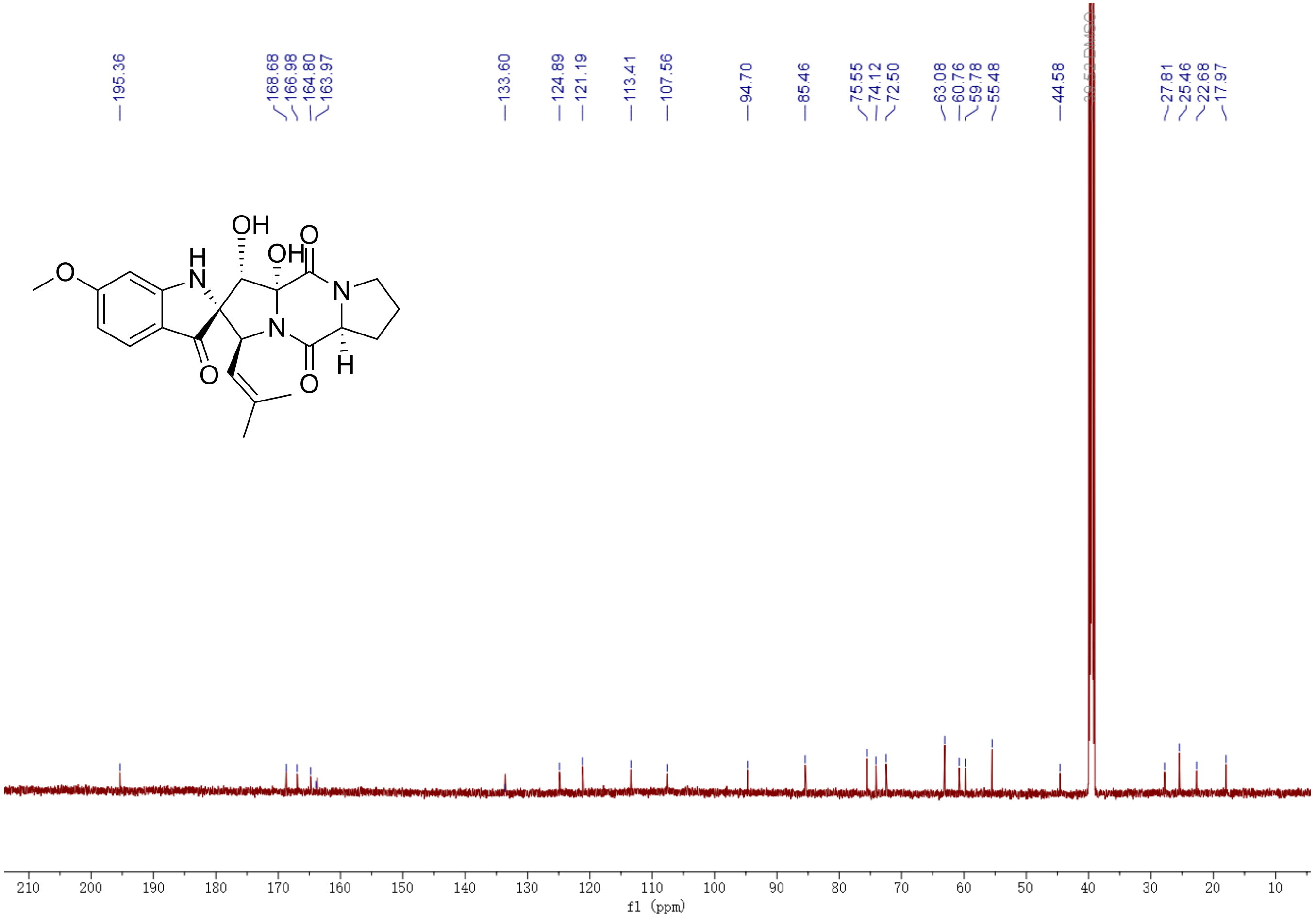

Fig. S20 ^{13}C NMR spectrum of **3**

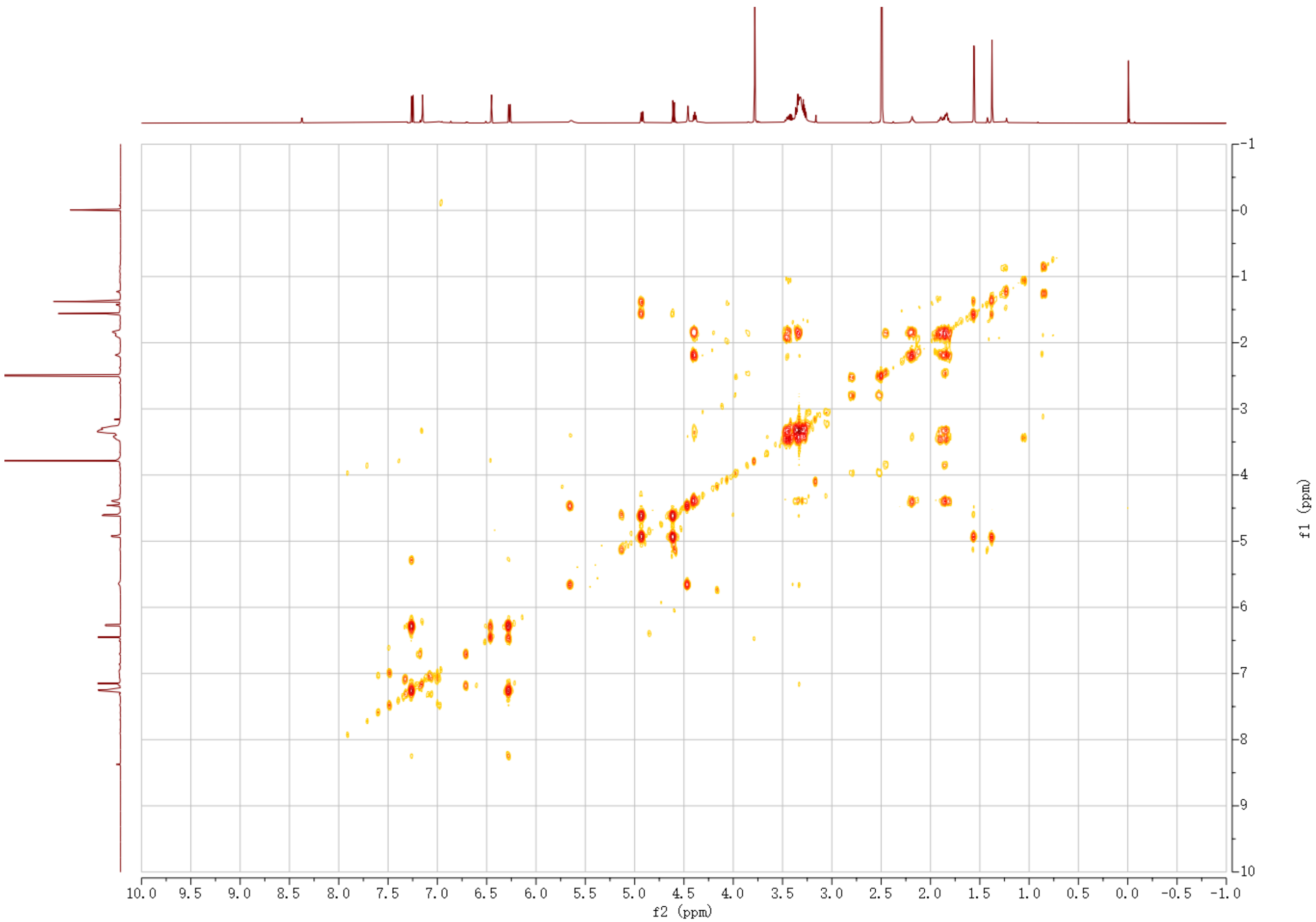

Fig. S21 ^{1}H-^{1}H COSY spectrum of **3**

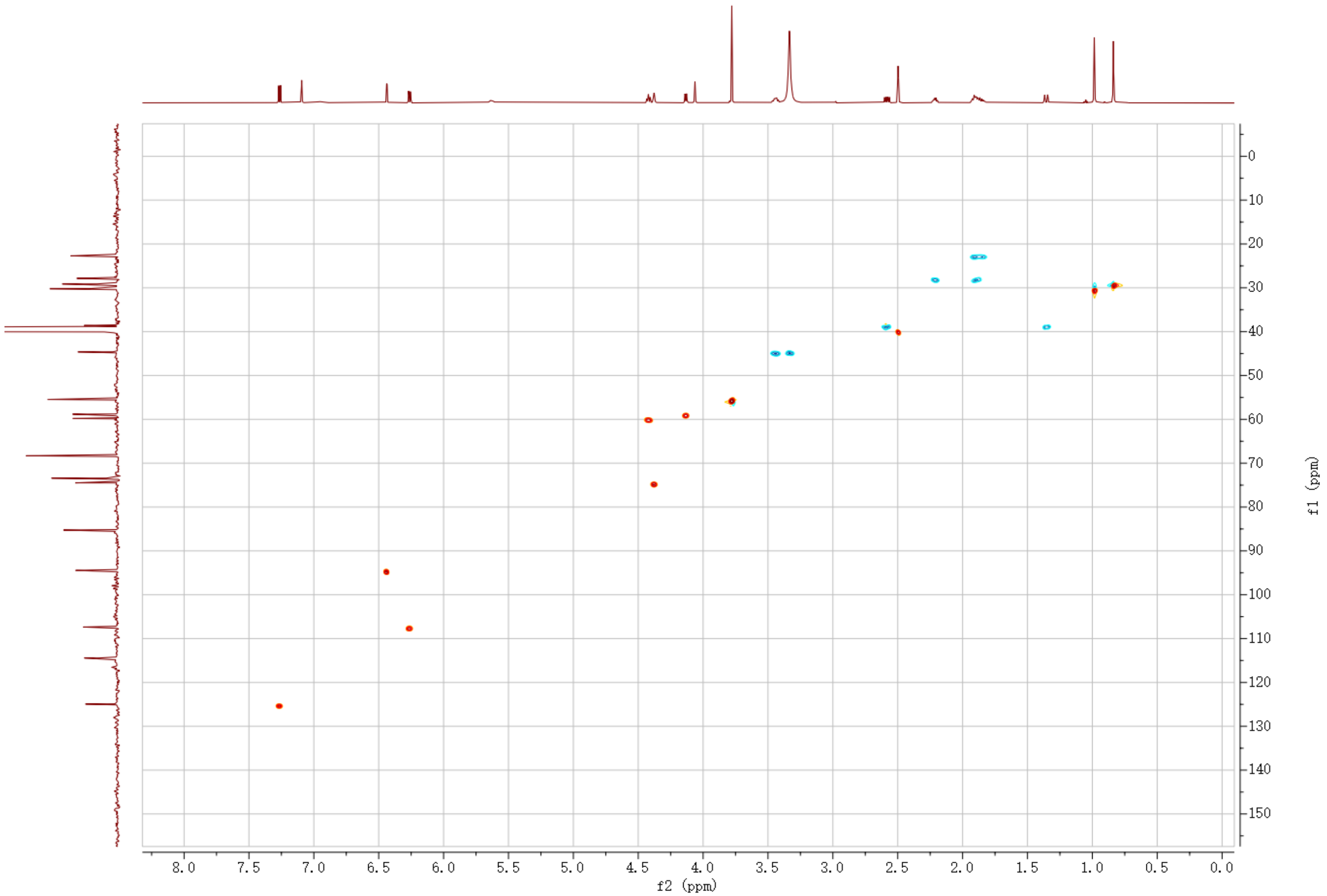

Fig. S22 HSQC spectrum of **3**

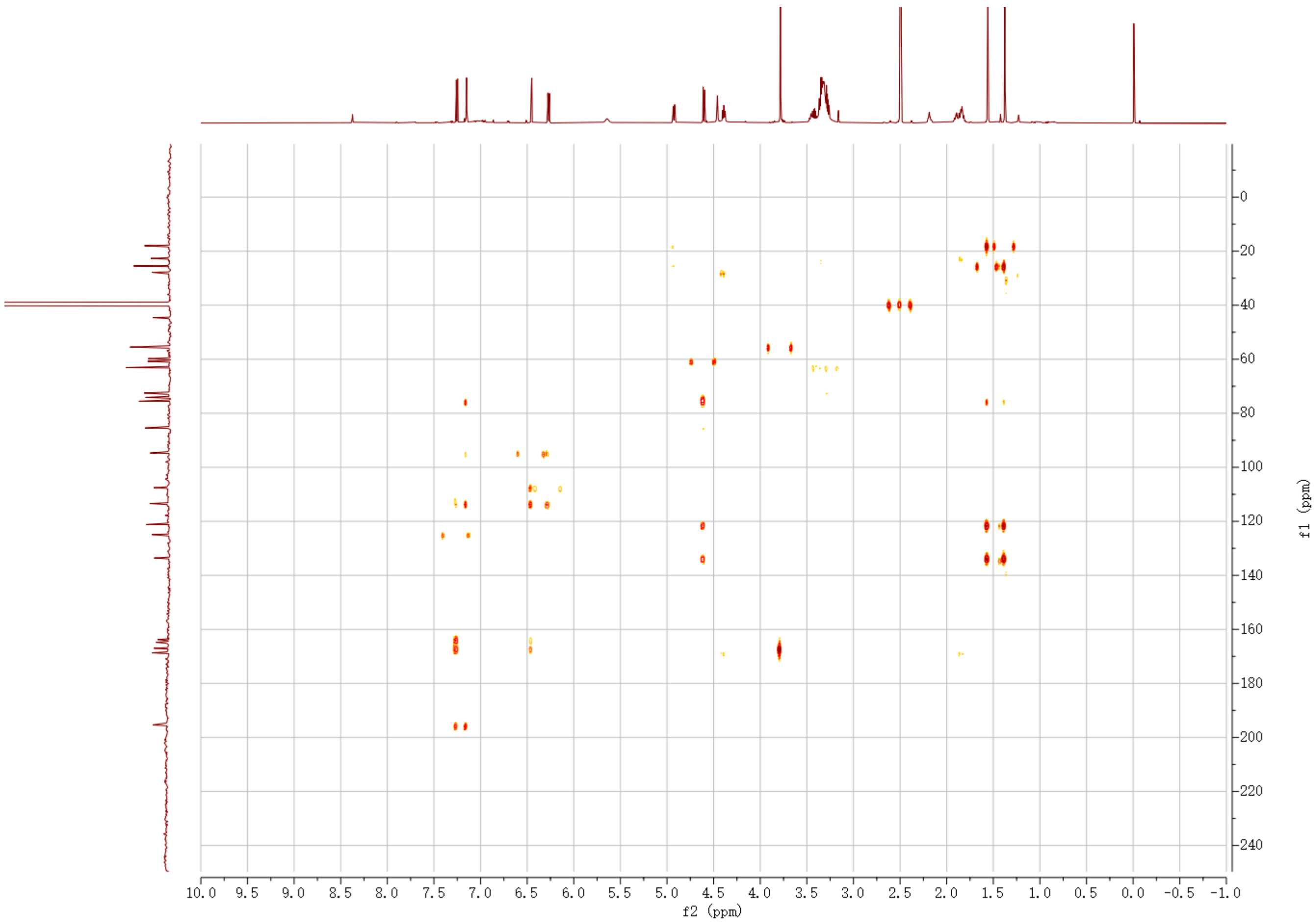

Fig. S23 HMBC spectrum of **3**

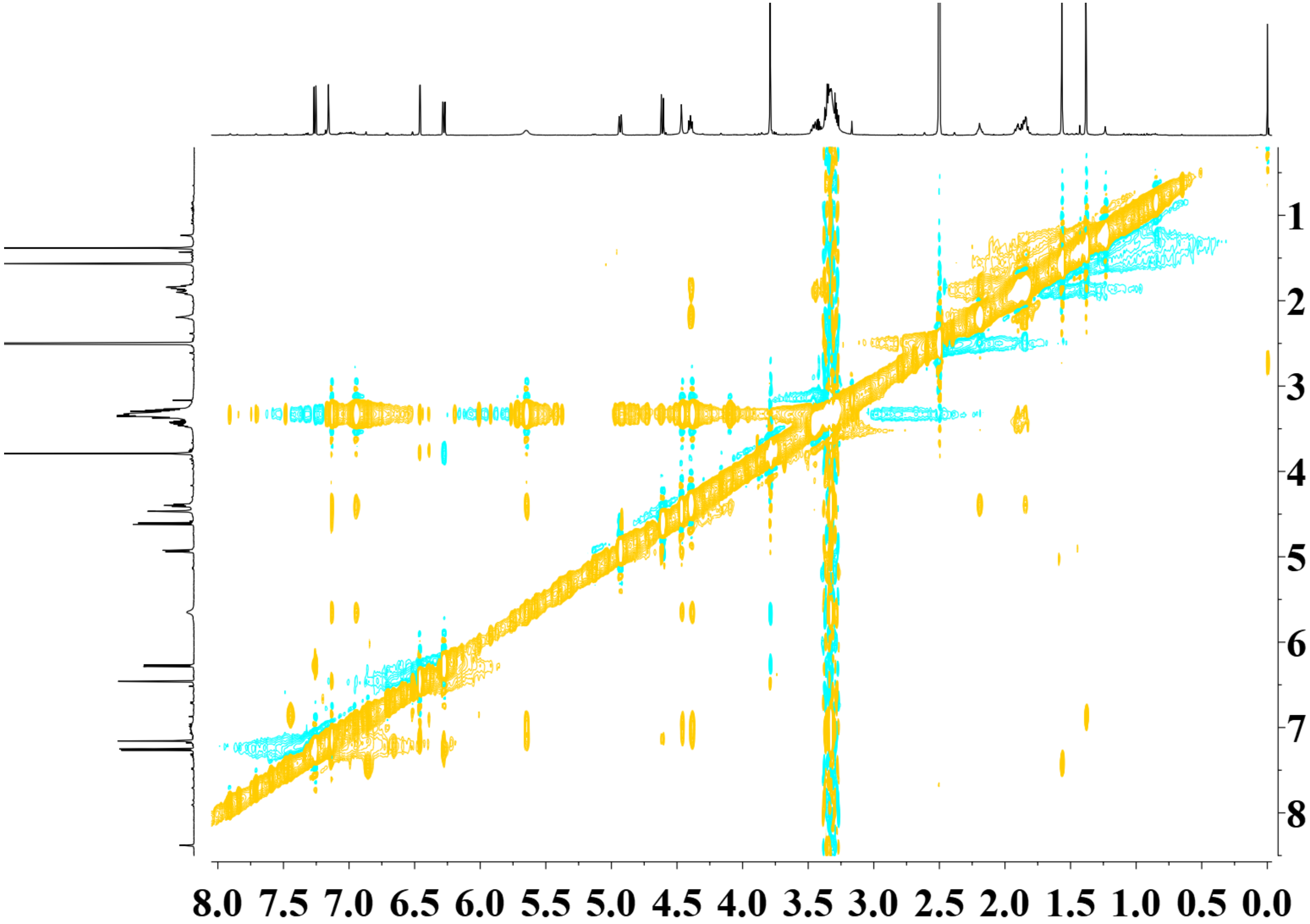

Fig. S24 NOESY spectrum of **3**

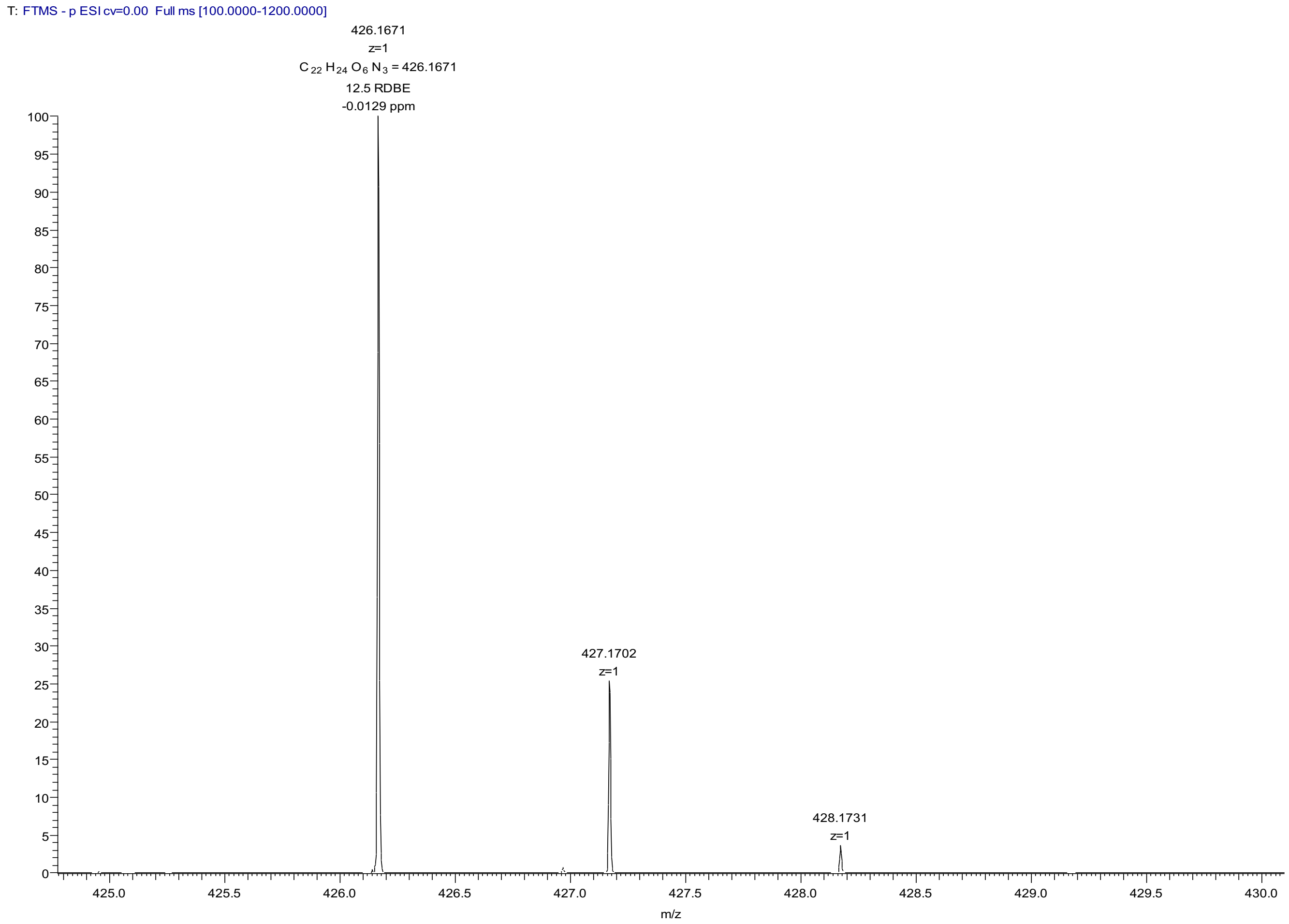

Fig. S25 HRESIMS spectrum of **3**

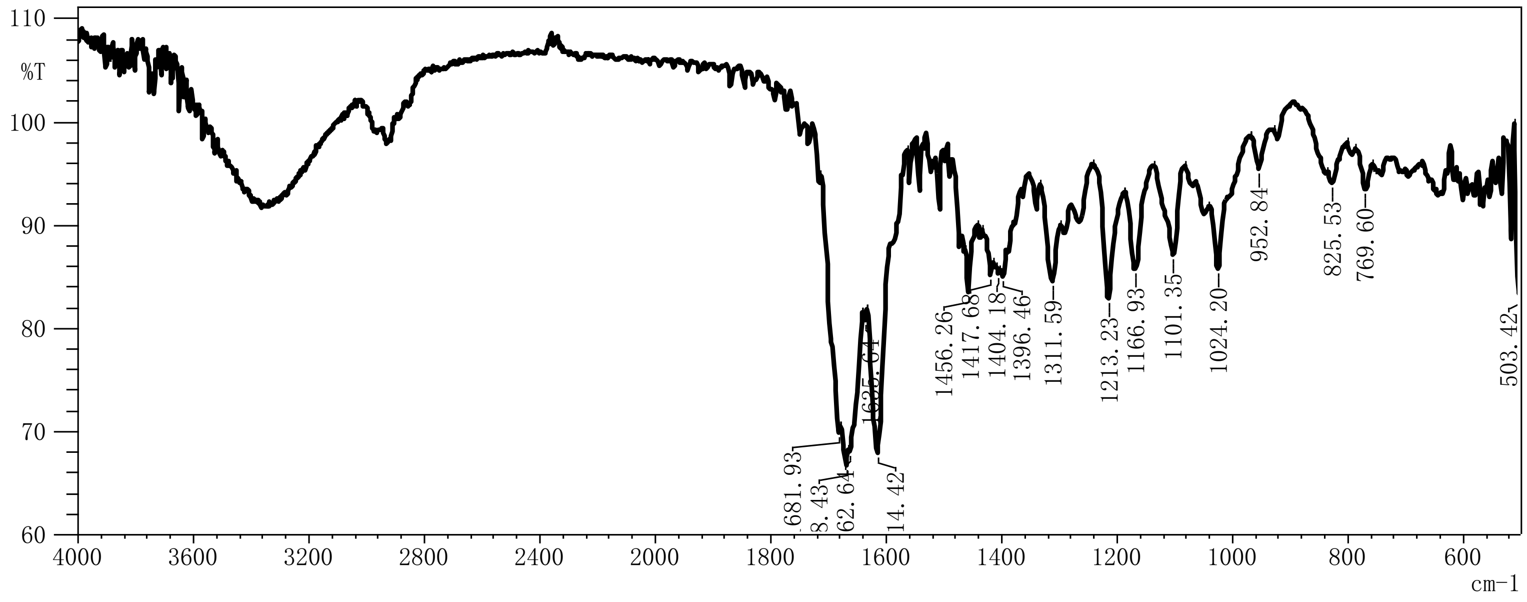

Fig. S26 IR spectrum of **3**

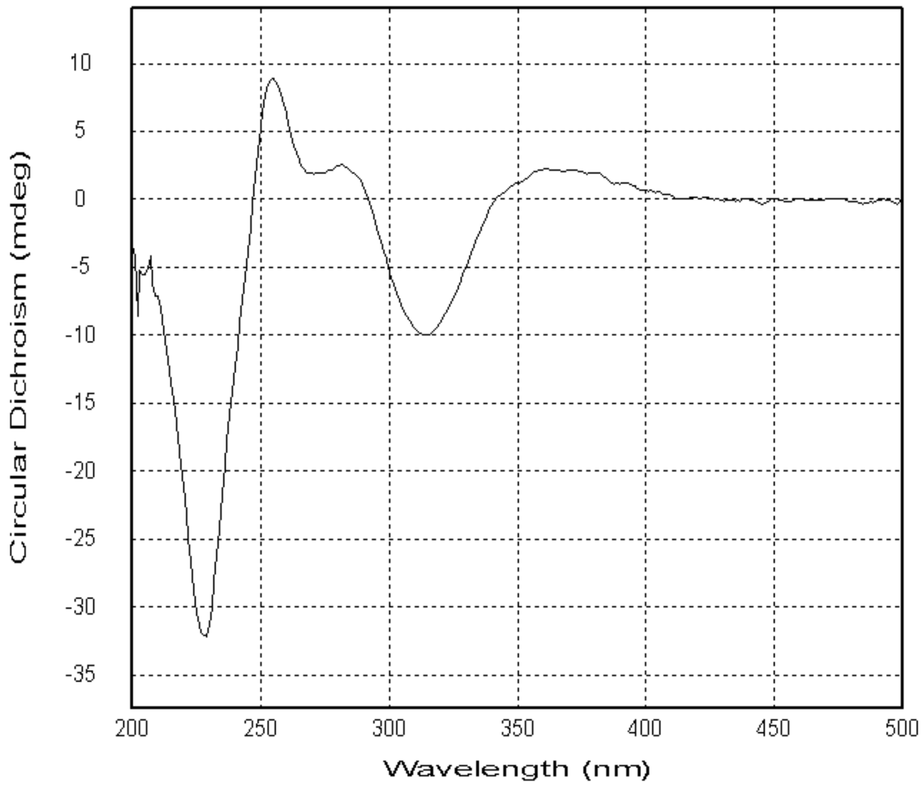

Fig. S27 Experimental CD spectrum of **3**

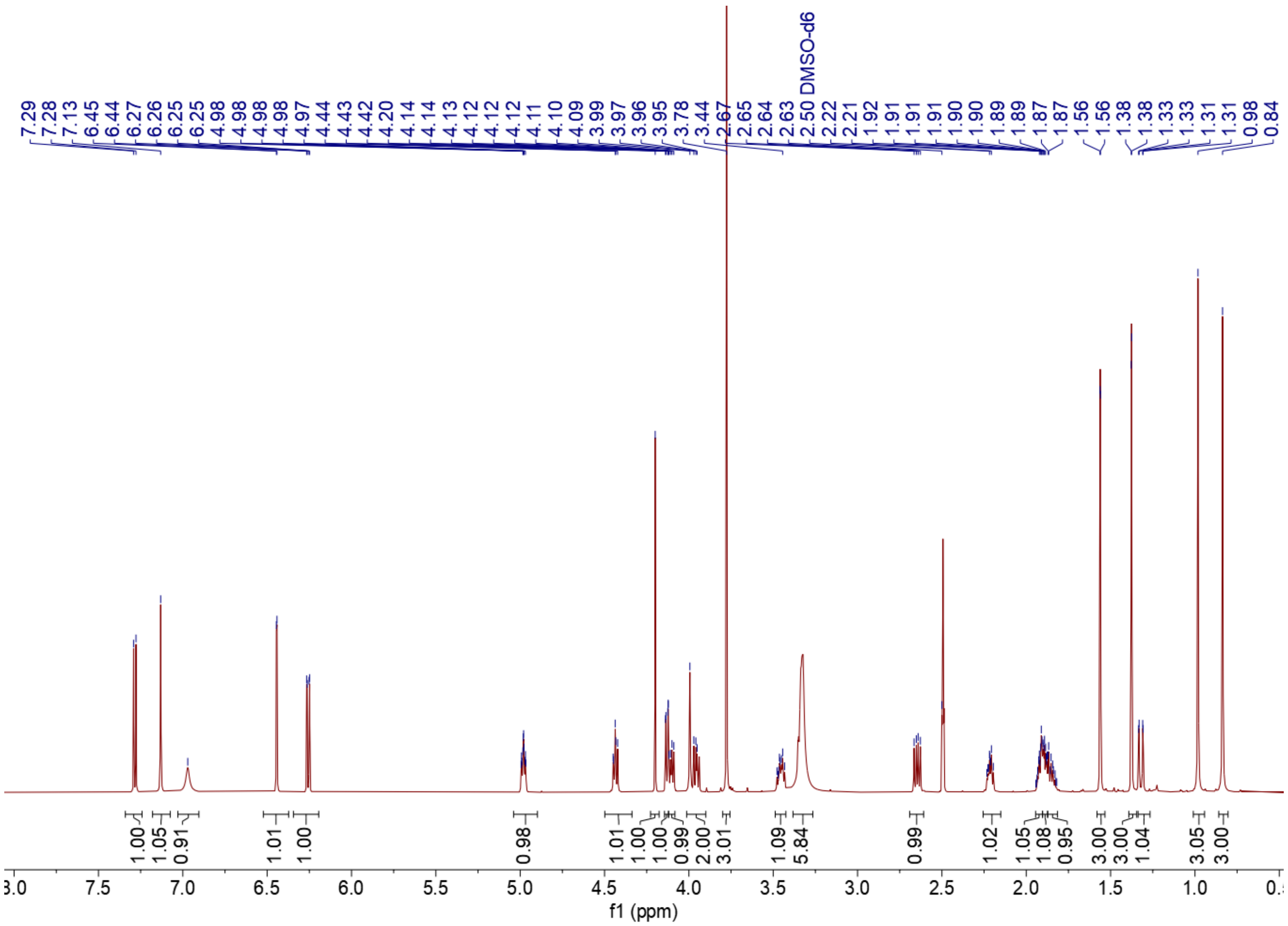

Fig. S28 ^{1}H NMR spectrum of **4**

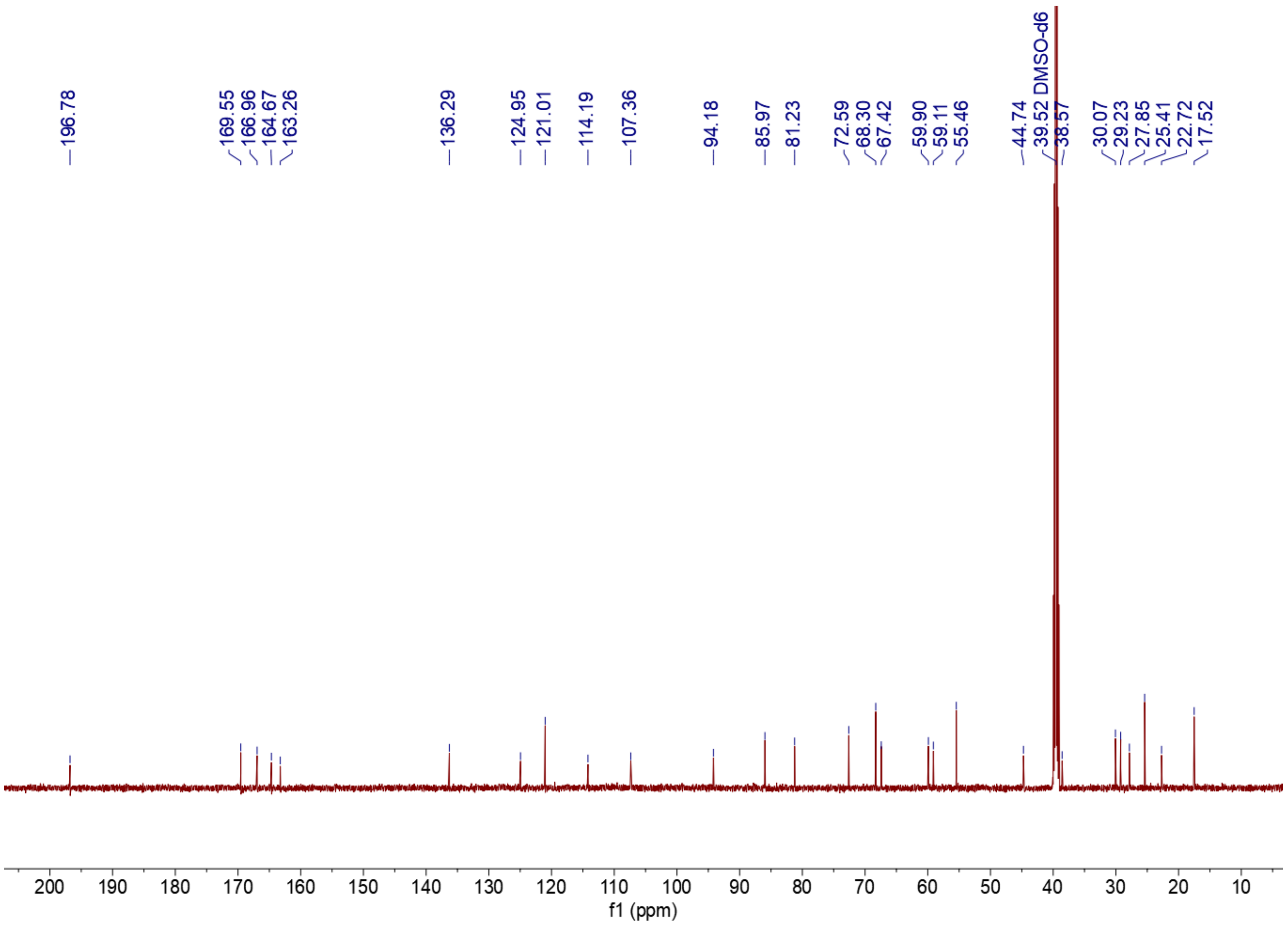

Fig. S29 ^{13}C NMR spectrum of **4**

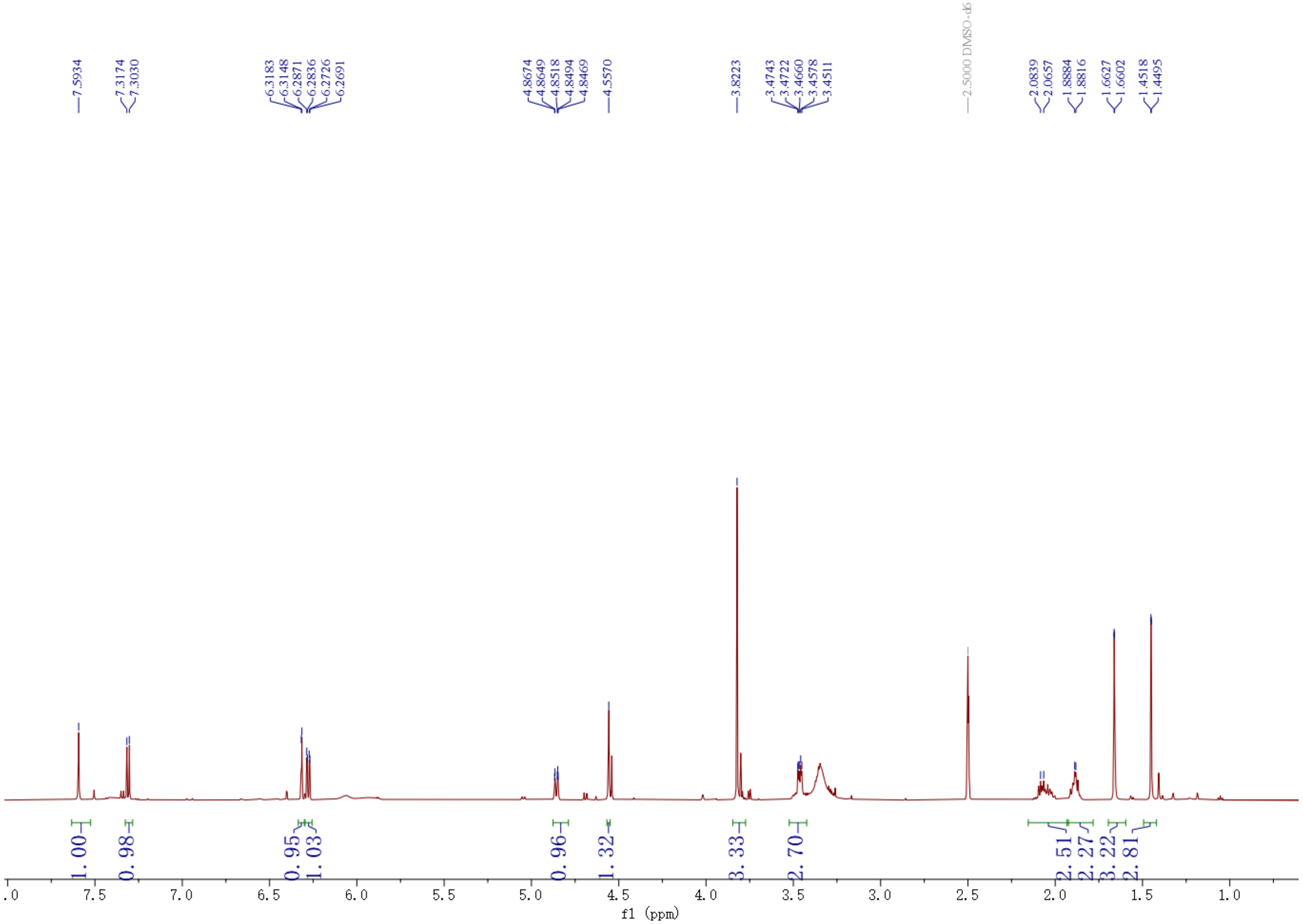

Fig. S30 ^{1}H NMR spectrum of **5**

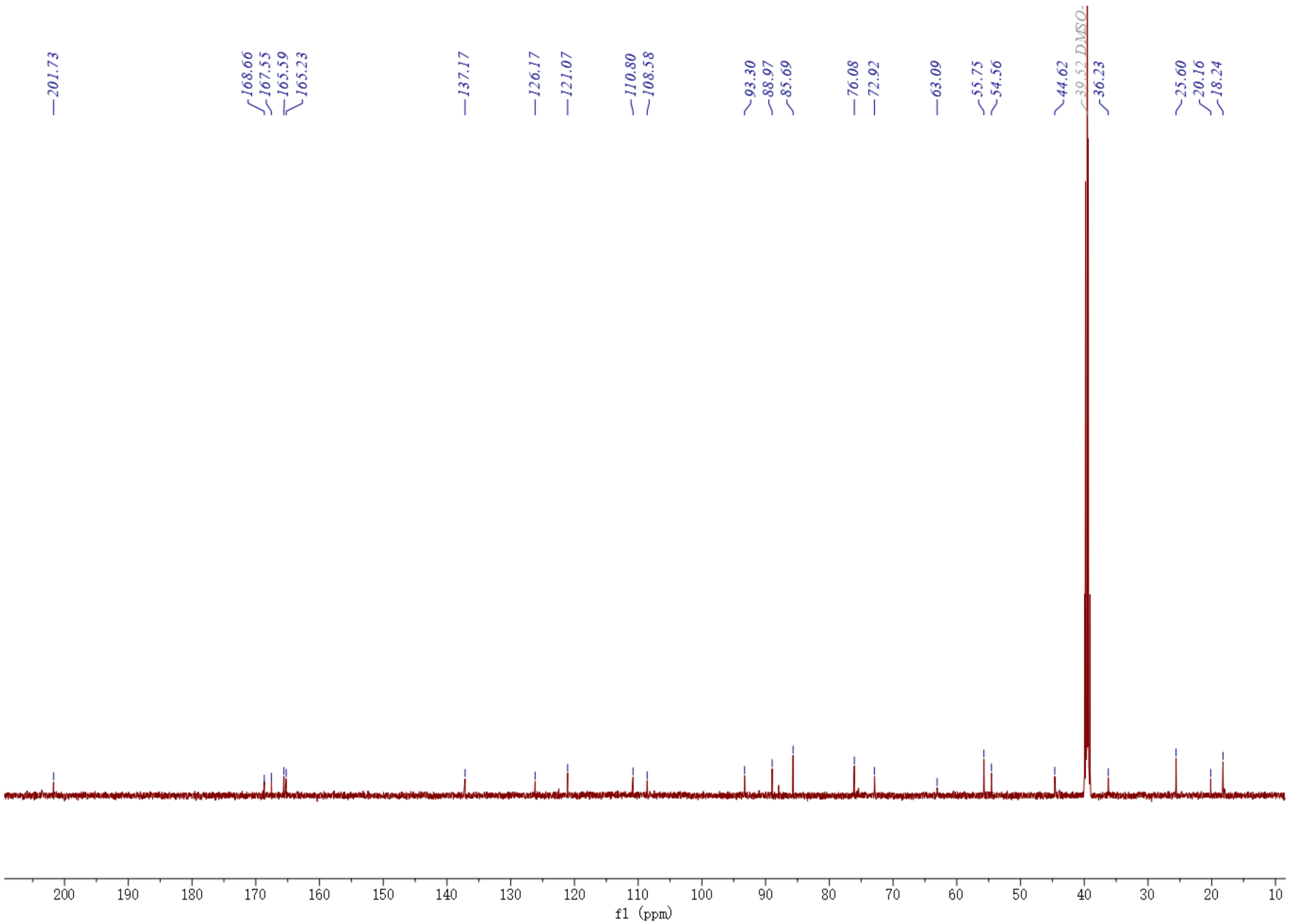

Fig. S31 ^{13}C NMR spectrum of **5**

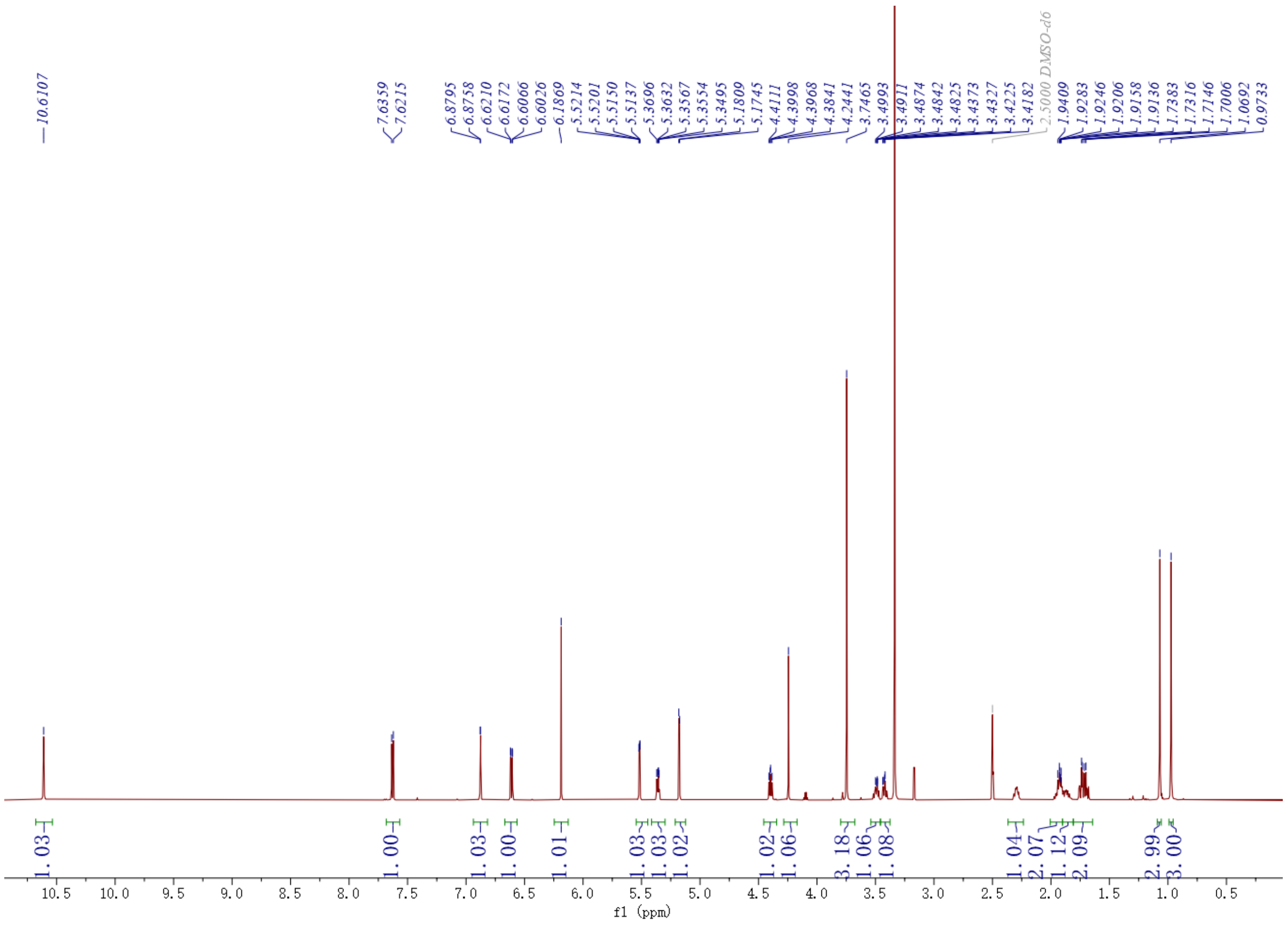

Fig. S32 ^{1}H NMR spectrum of **6**

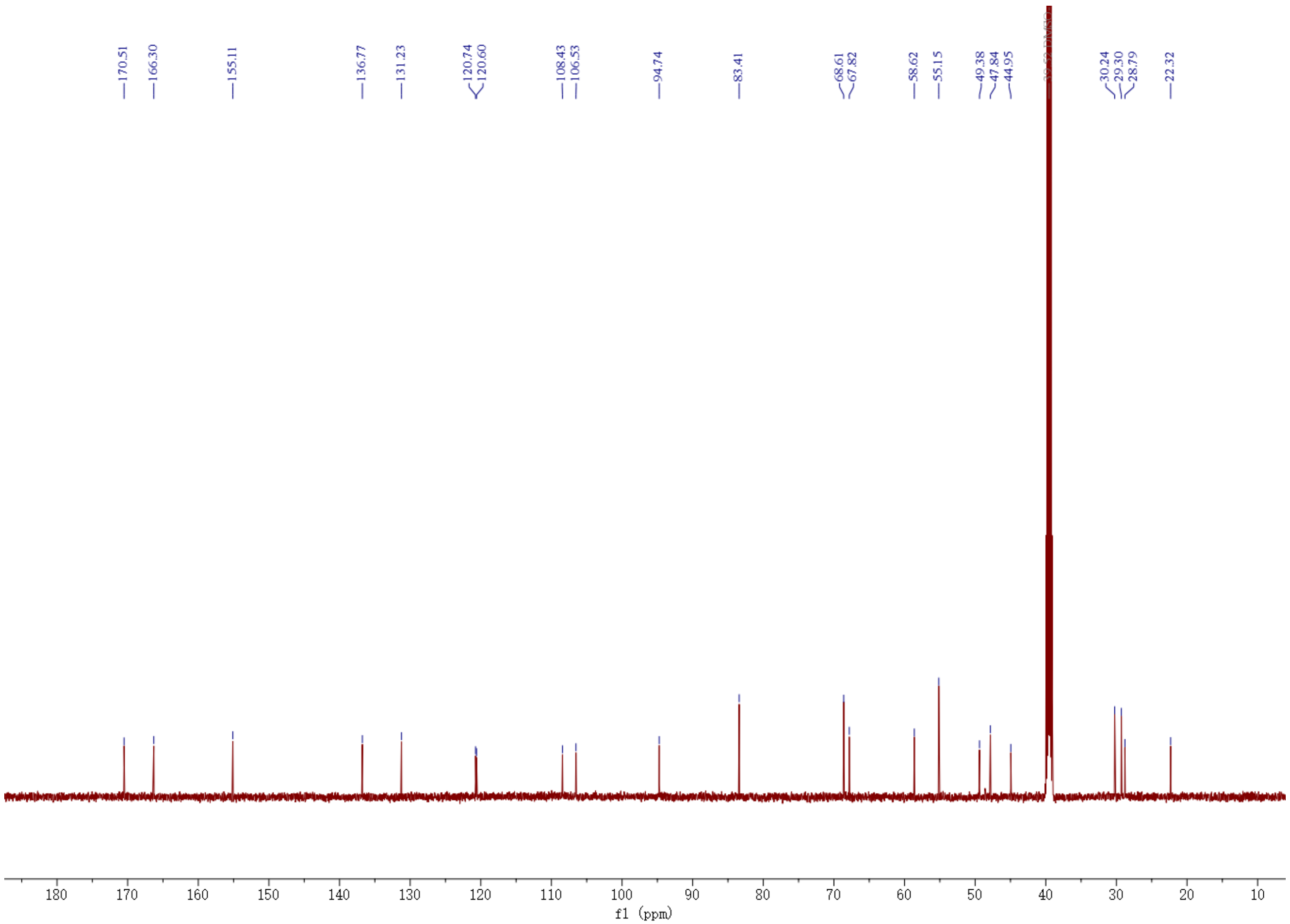

Fig. S33 ^{13}C NMR spectrum of **6**

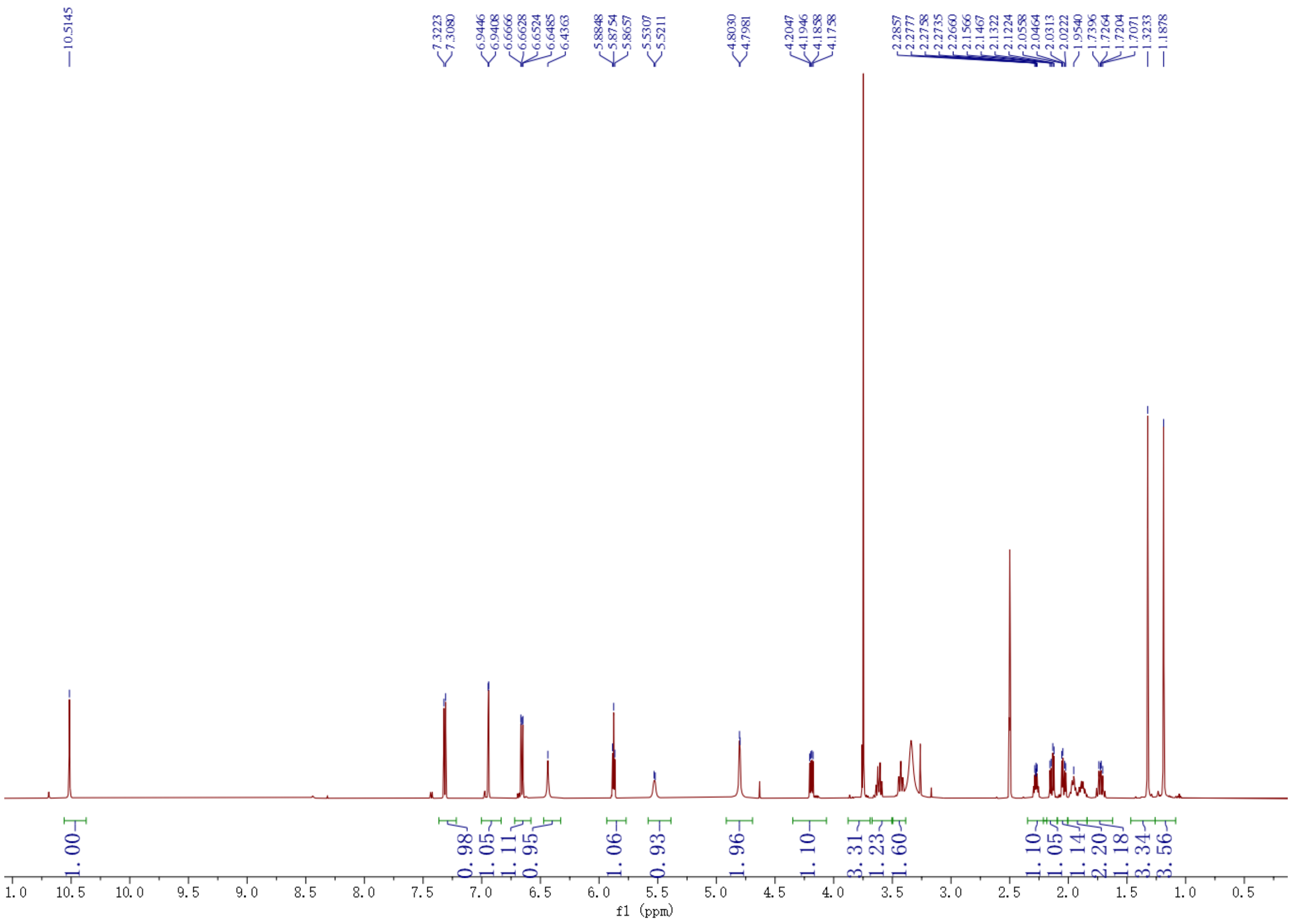

Fig. S34 ^{1}H-NMR spectrum of **7**

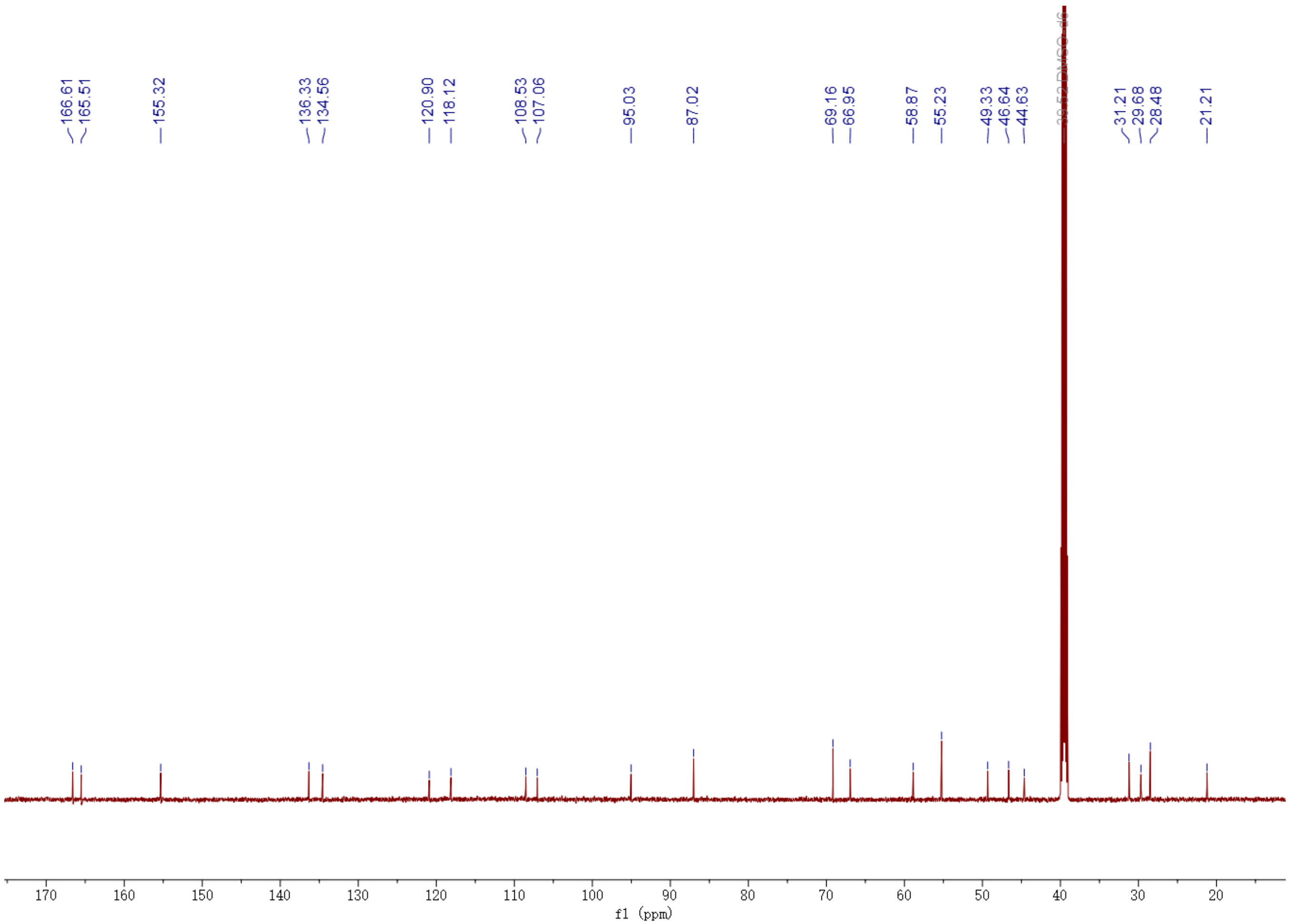

Fig. S35 ^{13}C-NMR spectrum of **7**

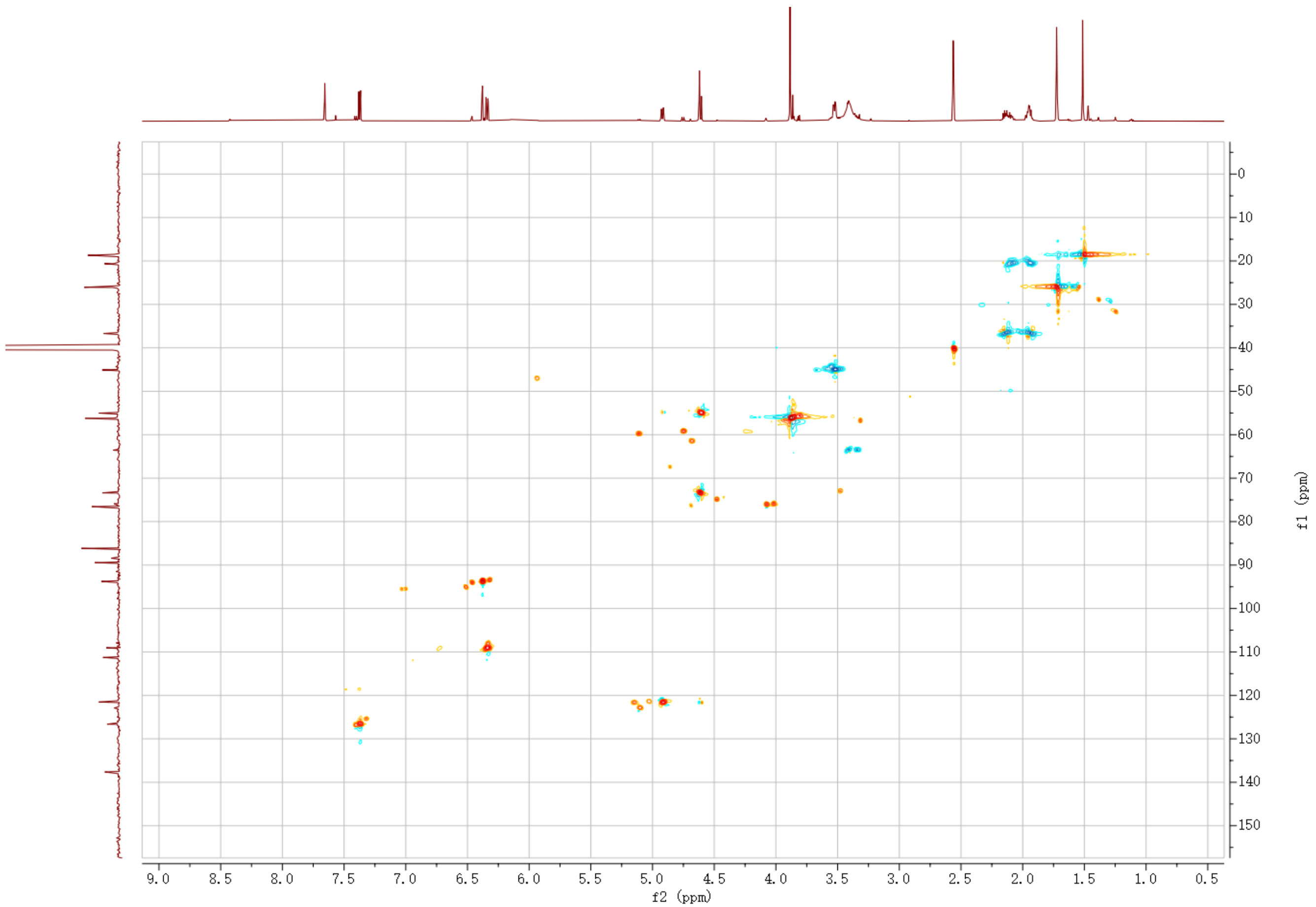

Fig. S36 HSQC spectrum of **5**

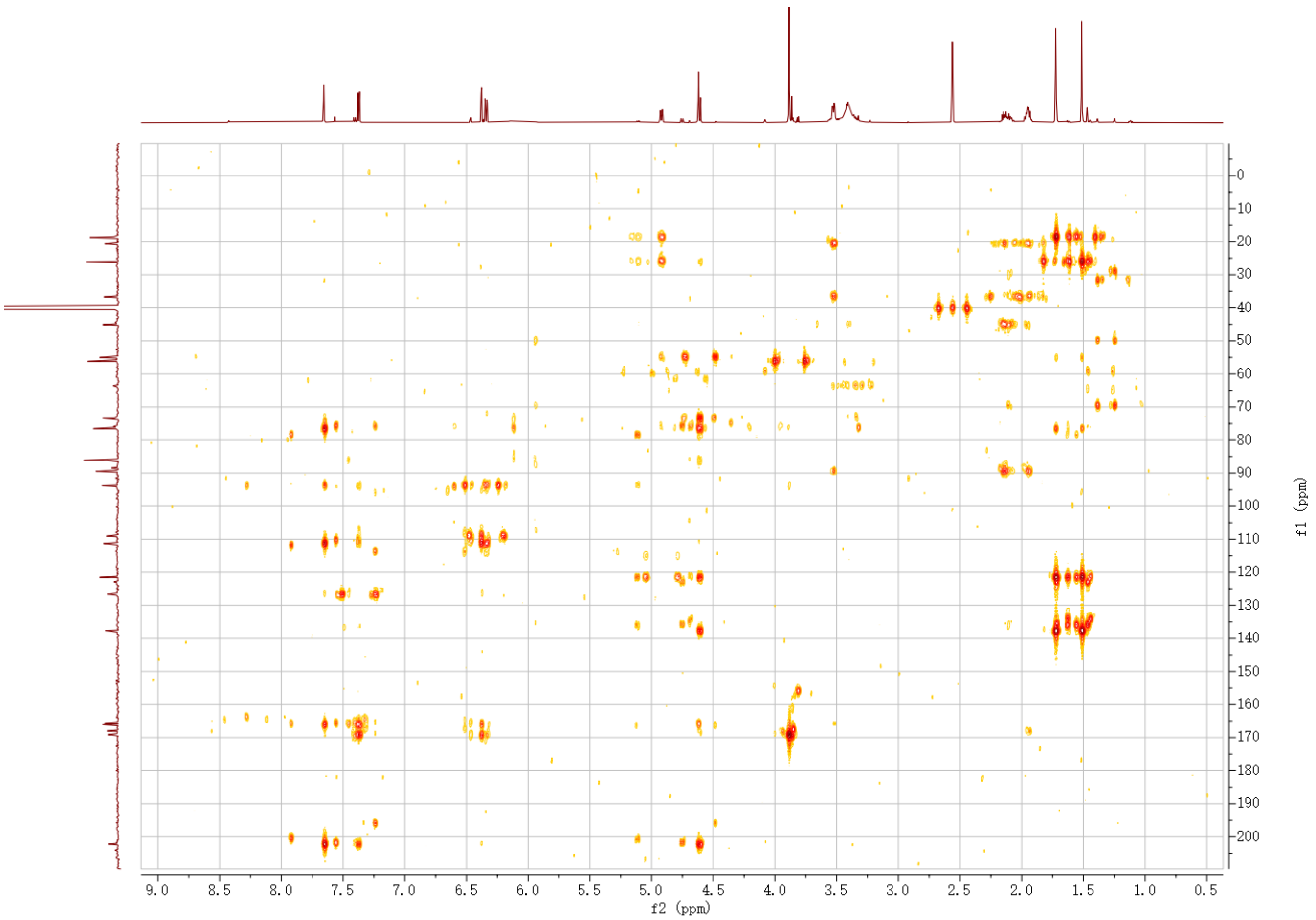

Fig. S37 HMBC spectrum of **5**

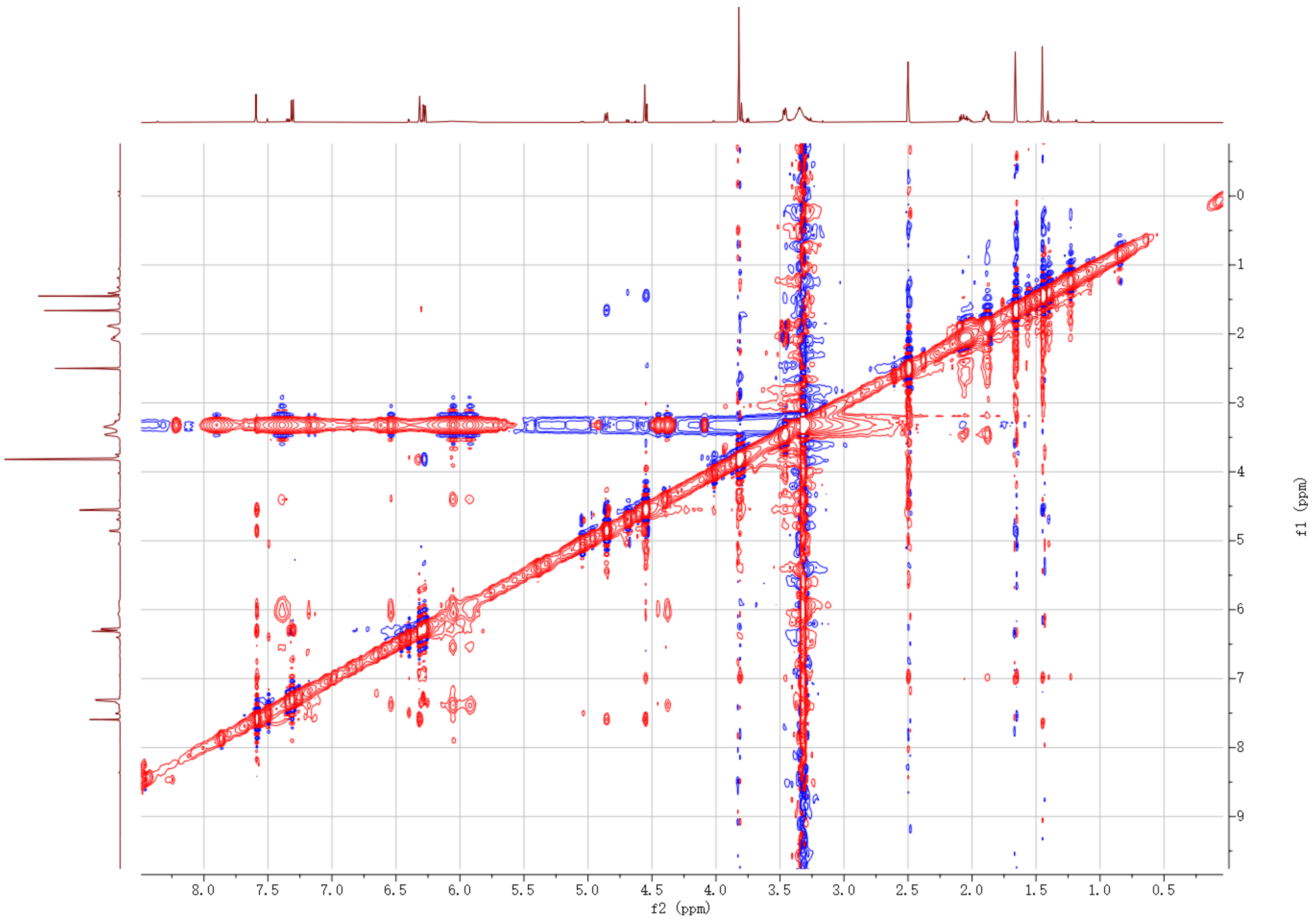

Fig. S38 NOESY spectrum of **5**

Table S1 DP4+ results of candidate configurations (2*R*,8*S*,9*R*,12*R*,18*S*)-**5**(Isomer 1**)** and (2*S*,8*S*,9*R*,12*R*,18*S*)-**5**(Isomer 2)

	A	B	C	D	E	F	G	H
1	Functional		Solvent?		Basis Set		Type of Data	
2	mPW1PW91		PCM		6-31+G(d,p)		Unscaled Shifts	
3								
4			Isomer 1	Isomer 2	Isomer 3	Isomer 4	Isomer 5	Isomer 6
5	sDP4+ (H data)		100.00%	0.00%	–	–	–	–
6	sDP4+ (C data)		100.00%	0.00%	–	–	–	–
7	sDP4+ (all data)		100.00%	0.00%	–	–	–	–
8	uDP4+ (H data)		100.00%	0.00%	–	–	–	–
9	uDP4+ (C data)		100.00%	0.00%	–	–	–	–
10	uDP4+ (all data)		100.00%	0.00%	–	–	–	–
11	DP4+ (H data)		100.00%	0.00%	–	–	–	–
12	DP4+ (C data)		100.00%	0.00%	–	–	–	–
13	DP4+ (all data)		100.00%	0.00%	–	–	–	–

Table S2 Linear correlation coefficients (R^2), root-mean-square deviation (RMSD) mean absolute deviation (MAD) and corrected mean absolute deviation (CMAD) analyses of the calculated and experimental NMR data

Candidate configurations	R^2	RMSD	MAD	CMAD
(2*R*,8*S*,9*R*,12*R*,18*S*)-**5**	0.9974	3.34	2.56	1.81
(2*S*,8*S*,9*R*,12*R*,18*S*)-**5**	0.9961	4.41	3.53	2.59

Table S3 Energy analyses of conformers (2*R*,8*S*,9*R*,12*R*,18*S*)-**5**a-(2*R*,8*S*,9*R*,12*R*,18*S*)-**5**b

NO.	3D comformers	Free energy		
		E (Hartree)	ΔE (Kcal/mol)	P(%)
(2*R*,8*S*,9*R*,12*R*,18*S*)-**5**a	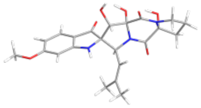	-1544.000934	0.001241	0.21
(2*R*,8*S*,9*R*,12*R*,18*S*)-**5**b	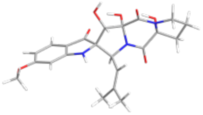	-1544.002175	0	0.79

Table S4 Experimental ^{13}C-NMR chemical shifts and GIAO isotropic magnetic shielding values calculated for PCM/mPW1PW91/6-31+G (d, p) geometries of (2*R*,8*S*,9*R*,12*R*,18*S*)-**5**.

NO.	Conformer (2*R*,8*S*,9*R*,12*R*,18*S*)-**5**a	Conformer (2*R*,8*S*,9*R*,12*R*,18*S*)-**5**b	Aver.	Exptl.	Scal.calcd.
C-2	78.1369	78.1428	78.13985	76.09	78.14155148
C-3	197.9702	197.4394	197.7048	201.7	197.5517244
C-3a	113.0223	111.8037	112.413	110.81	112.0615721
C-4	127.7538	126.2231	126.98845	126.17	126.5470166
C-5	103.4444	111.1061	107.27525	108.59	109.4847815
C-6	167.0752	167.3638	167.2195	168.67	167.3027284
C-7	97.7841	92.2484	95.01625	93.31	94.37070141
C-7a	161.8923	163.4941	162.6932	165.6	163.1551376
C-8	76.3641	76.2499	76.307	72.93	76.27406625
C-9	88.7239	88.767	88.74545	85.69	88.75787946
C-11	165.743	165.738	165.7405	167.55	165.7390581
C-12	93.5127	93.4545	93.4836	88.98	92.51594283
C-13	37.222	37.1128	37.1674	36.24	37.13590818
C-14	25.0654	25.0355	25.05045	20.16	25.04182724
C-15	47.4237	47.4539	47.4388	44.63	47.44750928
C-17	164.5728	164.4785	164.52565	165.23	164.4984551
C-18	60.2449	60.2084	60.22665	54.56	60.21612389
C-19	118.9369	118.648	118.79245	121.07	118.7091351
C-20	146.9339	147.2772	147.10555	137.17	147.2045531
C-21	19.5596	19.3903	19.47495	18.24	19.42612615
C-22	27.2495	27.4411	27.3453	25.6	27.40055487
C-23(-OCH_3)	56.3389	56.2705	56.3047	55.76	56.28497436

Table S5 Experimental ^{1}H-NMR chemical shifts and GIAO isotropic magnetic shielding values calculated for PCM/mPW1PW91/6-31+G (d, p) geometries of (2*R*,8*S*,9*R*,12*R*,18*S*)-**5**

NO.	Conformer (2*R*,8*S*,9*R*,12*R*,18*S*)-**5**a	Conformer (2*R*,8*S*,9*R*,12*R*,18*S*)-**5**b	Aver.	Exptl.	Scal.calcd.
NH	4.7833	4.9048	4.84405	7.59	4.879088971
H-4	8.0673	7.9203	7.9938	7.29	7.951407171
H-5	6.7642	6.7382	6.7512	6.25	6.743701949
H-7	6.6515	6.5917	6.6216	6.3	6.604354482
H-8	4.7183	4.7172	4.71775	4.54	4.717432775
H-13	2.2268	2.2292	2.228	2.05	2.228692128
	2.1473	2.155	2.15115	1.88	2.153370577
H-14	2.3749	2.3768	2.37585	2.01	2.376397935
	2.0626	2.062	2.0623	1.81	2.062126968
H-15	3.828	3.8206	3.8243	3.45	3.822165939
	3.5105	3.5082	3.50935	3.45	3.508686711
H-18	4.8028	4.8219	4.81235	4.53	4.817858184
H-19	5.1159	5.129	5.12245	4.83	5.126227864
H-21	1.570633333	1.5761	1.573366667	1.43	1.57494318
H-22	1.860233333	1.866166667	1.8632	1.65	1.864911094
H-23(-OCH_3)	3.9967	4.009066667	4.002883333	3.81	4.006449714
8-OH	2.4266	2.4646	2.4456	5.04	2.45655869
9-OH	8.9038	8.9816	8.9427	7.48	8.965136477
12-OH	5.7941	5.8072	5.80065	6.43	5.804427864

Table S6 Energy analyses of conformers (2*S*,8*S*,9*R*,12*R*,18*S*)-**5**a-(2*S*,8*S*,9*R*,12*R*,18*S*)-**5**h

NO.	3D comformers	Free energy		
		E (Hartree)	ΔE (Kcal/mol)	P(%)
(2*S*,8*S*,9*R*,12*R*,18*S*)-**5**a	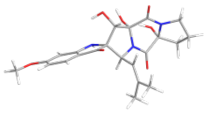	-1543.991711	0.002125	4.39
(2*S*,8*S*,9*R*,12*R*,18*S*)-**5**b	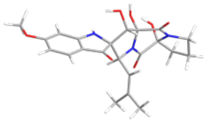	-1543.992647	0.001189	11.85
(2*S*,8*S*,9*R*,12*R*,18*S*)-**5**c	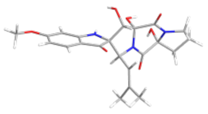	-1543.992582	0.001254	11.07
(2*S*,8*S*,9*R*,12*R*,18*S*)-**5**d	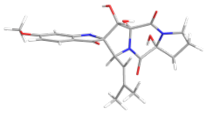	-1543.993395	0.000441	26.20
(2*S*,8*S*,9*R*,12*R*,18*S*)-**5**e	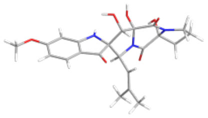	-1543.987618	0.006218	0.06
(2*S*,8*S*,9*R*,12*R*,18*S*)-**5**f	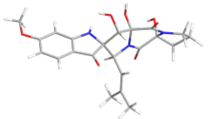	-1543.988628	0.005208	0.17
(2*S*,8*S*,9*R*,12*R*,18*S*)-**5**g	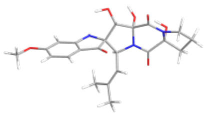	-1543.993836	0	41.81
(2*S*,8*S*,9*R*,12*R*,18*S*)-**5**h	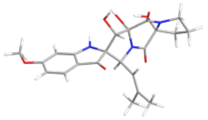	-1543.991722	0.002114	4.45

Table S7 Experimental ^{13}C-NMR chemical shifts and GIAO isotropic magnetic shielding values calculated for PCM/mPW1PW91/6-31+G (d, p) geometries of (2*S*,8*S*,9*R*,12*R*,18*S*)-**5**

NO.	Conformer 2*S*-**5**a	Conformer 2*S*-**5**b	Conformer 2*S*-**5**c	Conformer 2*S*-**5**d	Conformer 2*S*-**5**e	Conformer 2*S*-**5**f	Conformer 2*S*-**5**g	Conformer 2*S*-**5**h	Aver.	Exptl.	Scal.calcd.
C-2	80.083	79.6199	80.5638	79.9895	79.8094	80.1151	80.3866	77.6002	79.7709375	78.13985	80.07317804
C-3	191.431	190.945	192.0425	191.4192	192.2517	191.5322	191.3156	190.9033	191.4800625	197.7048	191.3668835
C-3a	114.4092	113.6745	114.2006	113.1925	114.1406	113.2806	114.3534	112.5969	113.7310375	112.413	113.8742533
C-4	125.9256	125.0309	125.7679	124.4192	125.7804	124.5908	126.1503	124.503	125.2710125	126.98845	125.4357494
C-5	103.3638	110.6835	101.61	108.9525	103.3109	111.1758	103.3587	108.6839	106.3923875	107.27525	105.7493151
C-6	165.5914	165.9325	165.4665	165.7272	165.5916	165.8571	165.6445	165.8539	165.7080875	167.2195	165.6879199
C-7	99.4787	93.5735	97.6102	91.5841	99.3738	92.8823	99.5202	90.0756	95.5123	95.01625	96.09142424
C-7a	158.7956	160.6179	158.646	160.5666	158.8883	160.9896	158.6931	160.4881	159.71065	162.6932	159.4952493
C-8	75.2632	75.4682	76.9467	77.2453	75.8892	76.0643	75.109	77.5974	76.1979125	76.307	76.03417729
C-9	88.0818	88.1258	88.1516	88.018	88.0473	88.1183	88.5391	87.506	88.0734875	88.74545	88.24363508
C-11	165.311	165.2024	164.4259	164.1554	166.4319	166.4936	165.8277	164.8323	165.335025	165.7405	165.0947639
C-12	94.2506	94.1966	93.2764	93.3593	92.3073	92.2073	93.3239	94.3107	93.4040125	93.4836	93.51358699
C-13	38.2109	38.1402	38.236	38.1033	39.8671	39.9293	37.6764	37.7509	38.4892625	37.1674	37.93701762
C-14	26.8841	26.8895	24.1969	24.2335	23.4374	23.4383	24.8904	27.0293	25.124925	25.05045	25.05813847
C-15	47.524	47.517	48.2448	48.3317	48.2418	48.1145	47.777	47.3602	47.888875	47.4388	47.91443137
C-17	163.4036	163.5382	163.9476	163.9539	163.8206	163.8071	163.3476	163.6667	163.6856625	164.52565	163.6131435
C-18	66.4749	66.351	64.7065	64.3855	65.8536	65.8893	66.1979	67.7819	65.955075	60.22665	65.65811168
C-19	117.644	117.5689	118.47	117.6682	118.3048	118.4984	118.3798	118.3404	118.1093125	118.79245	118.0732592
C-20	141.4412	141.4687	140.4569	141.1208	140.7135	141.0332	140.8998	140.6169	140.968875	147.10555	140.9874701
C-21	19.0649	18.9782	19.008	18.9184	19.0659	19.0574	19.0059	19.1997	19.0373	19.47495	18.99126156
C-22	27.308	27.3462	27.3441	27.3115	27.2299	27.3474	27.3022	27.418	27.3259125	27.3453	27.31993178
C-23(-OCH_3)	56.2287	56.027	56.0296	55.865	56.1116	56.0986	56.2065	55.902	56.058625	56.3047	56.06336669

Table S8 Experimental ^{1}H-NMR chemical shifts and GIAO isotropic magnetic shielding values calculated for PCM/mPW1PW91/6-31+G (d, p) geometries of (2*S*,8*S*,9*R*,12*R*,18*S*)-**5**

NO.	Conformer 2*S*-**5**a	Conformer 2*S*-**5**b	Conformer 2*S*-**5**c	Conformer 2*S*-**5**d	Conformer 2*S*-**5**e	Conformer 2*S*-**5**f	Conformer 2*S*-**5**g	Conformer 2*S*-**5**h	Aver.	Exptl.	Scal.calcd.
NH	4.5928	4.7156	4.959	5.1059	4.5943	4.7491	4.5975	6.0498	4.9205	7.59	4.849356545
H-4	8.038	7.8845	7.9776	7.846	8.0072	7.8735	8.0274	7.8543	7.9385625	7.29	7.949917579
H-5	6.8337	6.8148	6.7322	6.6676	6.7837	6.7494	6.8431	6.613	6.7546875	6.25	6.770652285
H-7	6.8224	6.7735	6.7074	6.621	6.8277	6.7951	6.8267	6.5408	6.739325	6.3	6.740339073
H-8	5.0111	5.0294	4.8369	4.8762	4.8916	4.9332	4.9801	4.9269	4.935675	4.54	4.941743951
H-13	3.0351	3.0468	2.225	2.2442	1.9719	1.998	2.0935	3.0621	2.459575	2.05	2.344831896
	2.1363	2.1341	2.1878	2.2153	2.1456	2.1631	2.2165	2.1346	2.1666625	1.88	2.195938684
H-14	1.9477	1.9577	2.3373	2.3484	2.347	2.3536	2.3628	1.9973	2.206475	2.01	2.273634986
	1.7217	1.737	2.0641	2.0742	1.9991	2.0117	2.034	1.7997	1.9301875	1.81	1.988434258
H-15	4.0241	4.0252	3.5468	3.5772	3.6387	3.6652	3.5253	3.9792	3.7477125	3.45	3.642975128
	3.545	3.5743	3.8053	3.8311	3.6674	3.6911	3.7691	3.5949	3.684775	3.45	3.748458742
H-18	4.909	4.9433	4.7646	4.7915	4.8992	4.9381	4.8965	4.9886	4.89135	4.53	4.864661152
H-19	5.4373	5.4597	5.4605	5.5583	5.4818	5.5052	5.409	5.4735	5.4731625	4.83	5.464141542
H-21	1.593966667	1.597833333	1.560366667	1.597166667	1.6058	1.5793	1.559066667	1.679866667	1.596670833	1.65	1.58075786
H-22	1.716133333	1.7132	1.728766667	1.722433333	1.699333333	1.7116	1.7231	1.787633333	1.725275	1.43	1.724910291
H-23(-OCH_3)	4.000733333	4.013966667	3.9851	3.9838	3.993866667	4.0131	4.0037	3.9722	3.995808333	3.81	3.996123686
8-OH	3.7957	3.6998	3.1043	3.0506	3.8642	3.8251	3.8011	2.5254	3.458275	5.04	3.458441098
9-OH	4.1003	4.0631	6.0839	6.1677	3.8851	3.8887	4.1072	4.5285	4.6030625	7.48	4.878498872
12-OH	5.4267	5.4591	2.7672	2.8104	2.2161	2.219	5.1201	5.0961	3.8893375	6.43	4.30067574
